# The burden of disease in Greece, health loss, risk factors, and health financing, 2000–16: an analysis of the Global Burden of Disease Study 2016

**DOI:** 10.1016/S2468-2667(18)30130-0

**Published:** 2018-07-25

**Authors:** Global Burden of Disease 2016 Greece Collaborators, Stefanos Tyrovolas, Nick J Kassebaum, Andy Stergachis, Haftom N Abraha, François Alla, Sofia Androudi, Mate Car, Vanessa Chrepa, Nancy Fullman, Thomas Fürst, Josep Maria Haro, Simon I Hay, Mihajlo B Jakovljevic, Jost B Jonas, Ibrahim A Khalil, Jacek A Kopec, Helena Manguerra, Ira Martopullo, Ali Mokdad, Lorenzo Monasta, Emma Nichols, Helen E Olsen, Salman Rawaf, Robert Reiner, Andre M N Renzaho, Luca Ronfani, Maria Dolores Sanchez-Niño, Benn Sartorius, Dayane G A Silveira, Vasiliki Stathopoulou, Emil Stein Vollset, Konstantinos Stroumpoulis, Monika Sawhney, Roman Topor-Madry, Fotis Topouzis, Miguel Tortajada-Girbés, Miltiadis Tsilimbaris, Nikolaos Tsilimparis, Dimitrios Valsamidis, Job F M van Boven, Francesco S Violante, Andrea Werdecker, Ronny Westerman, Harvey A Whiteford, Charles D A Wolfe, Mustafa Z Younis, Georgios A Kotsakis

## Abstract

**Background:**

Following the economic crisis in Greece in 2010, the country's ongoing austerity measures include a substantial contraction of health-care expenditure, with reports of subsequent negative health consequences. A comprehensive evaluation of mortality and morbidity is required to understand the current challenges of public health in Greece.

**Methods:**

We used the results of the Global Burden of Diseases, Injuries, and Risk Factors Study (GBD) 2016 to describe the patterns of death and disability among those living in Greece from 2000 to 2010 (pre-austerity) and 2010 to 2016 (post-austerity), and compared trends in health outcomes and health expenditure to those in Cyprus and western Europe. We estimated all-cause mortality from vital registration data, and we calculated cause-specific deaths and years of life lost. Age-standardised mortality rates were compared using the annualised rate of change (ARC). Mortality risk factors were assessed using a comparative risk assessment framework for 84 risk factors and clusters to calculative summary exposure values and population attributable fraction statistics. We assessed the association between trends in total, government, out-of-pocket, and prepaid public health expenditure and all-cause mortality with a segmented correlation analysis.

**Findings:**

All-age mortality in Greece increased from 944·5 (95% uncertainty interval [UI] 923·1–964·5) deaths per 100 000 in 2000 to 997·8 (975·4–1018) in 2010 and 1174·9 (1107·4–1243·2) in 2016, with a higher ARC after 2010 and the introduction of austerity (2·72% [1·65 to 3·74] for 2010–16) than before (0·55% [0·24 to 0·85] for 2000–10) or in western Europe during the same period (0·86% [0·54 to 1·17]). Age-standardised reduction in ARC approximately halved from 2000–10 (−1·61 [95% UI −1·91 to −1·30]) to 2010–16 (−0·87% [–2·03 to 0·20]), with post-2010 ARC similar to that in Cyprus (−0·86% [–1·4 to −0·36]) and lower than in western Europe (−1·14% [–1·48 to −0·81]). Mortality changes in Greece coincided with a rapid decrease in government health expenditure, but also with aggregate population ageing from 2010 to 2016 that was faster than observed in Cyprus. Causes of death that increased were largely those that are responsive to health care. Comparable temporal and age patterns were noted for non-fatal health outcomes, with a somewhat faster rise in years lived with disability since 2010 in Greece compared with Cyprus and western Europe. Risk factor exposures, especially high body-mass index, smoking, and alcohol use, explained much of the mortality increase in Greek adults aged 15–49 years, but only explained a minority of that in adults older than 70 years.

**Interpretation:**

The findings of increases in total deaths and accelerated population ageing call for specific focus from health policy makers to ensure the health-care system is equipped to meet the needs of the people in Greece.

**Funding:**

Bill & Melinda Gates Foundation.

## Introduction

Greece entered an austerity programme in 2010 as part of a financial bailout imposed by the European Commission and the European Central Bank in coalition with the International Monetary Fund.^1^ Its recession has been compared with the USA's great depression of 1929–39, with most of the budget allocated to debt payoff, and contraction of national gross domestic product (GDP).^2^ Compounded by a shrinking economy, health expenditure in Greece also proportionally shrank from 9·8% of GDP in 2008 to 8·1% in 2014.^2^, ^3^, ^4^ Reduced health spending, required as part of the austerity programme, has been criticised for not containing specific provisions to safeguard the National Health System,^4^, ^5^ a system instituted in the 1980s as part of the national programme of compulsory social insurance and through which most residents of Greece receive care. By contrast, health expenditure within the EU rose from 9·4% of GDP in 2008 to 10% in 2014.^6^

Following the onset of austerity measures, multiple reports noted adverse health trends, increasing out-of-pocket health expenditure, and unmet health-care needs.^7^, ^8^, ^9^ According to the Hellenic Centre for Disease Control and Prevention, tuberculosis rates have risen among native-born Greeks.^8^ HIV incidence nearly doubled from 2010 to 2012, reaching 10·4 per 100 000 population and prompting reinstatement of syringe distribution programmes; subsequently, HIV incidence decreased from 2012 to 2016, back to 5·7 per 100 000.^10^ Increasing rates of major depression and suicidality have been documented,^4^, ^11^ along with stagnation in maternal, infant, and child mortality.^3^, ^12^, ^13^

**Research in context**

**Evidence before this study**

Following the global financial crisis, Greece entered into an austerity programme in 2010 that has substantially affected health expenditures. Reports from the Hellenic Statistical Authority, the European Centers for Disease Control, and published studies have documented untoward effects on public health following the onset of austerity measures. However, these studies have generally been limited in scope, including being limited to examining only a few causes of illness or mortality, examining only official statistics, and not continuing beyond the first few years of austerity. To better understand the current state of public health in Greece, its trajectory pre-2010 and post-2010, and the broader impacts of financial crisis and austerity on health requires comprehensive evaluation of multiple health-related domains, including all-cause and cause-specific mortality, non-fatal health loss, risk factors, health spending, and demographics.

**Added value of this study**

This study expands on what is known about the effects of the austerity-driven reductions in national health spending on population health in Greece by utilising data from the most comprehensive effort to date to estimate summary measures of global population health—the Global Burden of Diseases, Injuries, and Risk Factors (GBD) initiative. Previous research has focused on the reporting of short-term mortality and morbidity trends that were observed within the first years of the implementation of the austerity programme that limits their sensitivity to detect adverse health outcomes with longer incubation times. In addition to assessing risk factors for the aforementioned health changes, this study investigates the temporal linkage between health financing and health loss until 2015. We did a comprehensive analysis of annual changes in population health in Greece from 2000 to 2016 using regional comparators that allowed us to capture region-specific divergent temporal trends, and we assessed these trends in conjunction with population structure data and health financing measures. The combination of these data sources enabled us to identify evidence of a disproportionate decrement in the health of Greeks as compared with regional populations from 2000 to 2010 (pre-austerity era) to those from 2010 to 2016 (post-austerity era), which was concordant with decreases in national health spending. Further comparison of crude and age-standardised mortality rates indicated accelerated population ageing in Greece compared with Cyprus, with major population shrinkage in the 15–34 years that might be associated to indirect effects of the crisis, such as longstanding unemployment, reduced wages, and stringent taxation schedules.

**Implications of all the available evidence**

These findings point to multifaceted effects of the financial crisis in Greece: direct effects of reduced health spending on mortality and to a lesser extent morbidity age and temporal trends, and indirect effects related to a major shift in demographics with population ageing emerging as a public health concern. The increase in total deaths in children younger than 5 years and older adults with increase in causes sensitive to resource availability (eg, access to screening and urgent care) suggest that the health system requires substantial restructuring to cope with the effects that the financial crisis has had on resource availability, resource allocation, and population structure.

Despite the temporal relationship with austerity measures, these reports were criticised for not being supported by national estimate data,^14^ or being only partly associated with the economic downturn.^15^ Furthermore, some published communications reported less dramatic health changes in Greece, including actual improvements in cardiovascular mortality.^16^, ^17^ The discordance of estimates derived from different data sources, such as between the Hellenic Statistical Authority (ELSTAT) and the EU's statistical office (EUROSTAT), has been further suggested as an obstacle to obtaining pragmatic estimates for temporal changes in health indicators in Greece.^18^

As a sovereign nation neighbouring Greece that shares the Greek ethnicity (with a smaller fraction of Turkish Cypriots, Armenians, and Maronites), language, and socioeconomic structure at large, Cyprus represents as close a direct comparator to Greece as exists in western Europe. Although in former years large proportions of the population adhered to a Mediterranean diet, dietary habits are changing rapidly, with increasing obesity, persistent smoking, and alcohol use despite public health efforts to reduce consumption. Similar to Greece, a steep rise in HIV was documented in Cyprus post-2010,^19^ but the prevalence and trends of other conditions in Cyprus have been much less explored. Cyprus also felt the profound impacts from the recession and entered a financial bailout programme in the 2012–13 period in the aftermath of the Greek financial crisis, although recovery was faster than in Greece.^19^

Documenting of the effect of diseases on population health is very demanding from the standpoint of both surveillance and analytical methods. The only comprehensive effort to quantify global population health is the Global Burden of Diseases, Injuries, and Risk Factors Study (GBD).^20^ GBD is therefore the natural platform for comparison between different locations. In this report, we use the GBD 2016 results to explore temporal trends in health loss, risk factors, and health financing in Greece from 2000 to 2016, comparing with those of Cyprus and western Europe overall.

## Methods

### Overview and metrics

GBD 2016 quantified multiple measures of health loss for 333 causes and 84 risk factors for each of 195 countries and territories, 23 age groups, and both sexes from 1990 to 2016.^21^ For the present analysis, we compared Greece, Cyprus (estimates refer to the Republic of Cyprus only), and the GBD region of western Europe (Andorra, Austria, Belgium, Cyprus, Denmark, Finland, France, Germany, Greece, Iceland, Ireland, Israel, Italy, Luxembourg, Malta, the Netherlands, Norway, Portugal, and Spain). Our comparisons are primarily for both sexes combined and for wider-ranging age groups than those used in the analysis.

We compared rates across locations to control for population size, age standardisation to control for age structure, and comparative rankings and disease rates from more granular age groups to examine specific causes and risk factors more closely. We compared trends using the annualised rate of change (ARC), computed as the final estimates, divided by initial estimates, then divided by number of years, using the natural log transformation of this figure. Detailed methods for the overall study and for each specific cause and risk are available in the GBD 2016 summary publications,^21^, ^22^, ^23^, ^24^, ^25^ each of which is GATHER compliant. The next sections are brief descriptions of methods for deriving each of the GBD metrics included in the present analysis; these metrics include deaths, health expenditure, years lived with disability (YLDs), and summary exposure values (SEV) for risk factors.

The GBD study's protocol has been approved by the research ethics board at the University of Washington (UW). The GBD study shall be conducted in full compliance with UW policies and procedures, as well as applicable federal, state, and local laws.

### All-cause mortality and causes of death

Mortality estimation methods are described in detail elsewhere.^21^, ^22^ Briefly, all-cause mortality was estimated primarily from vital registration data, adjusted for completeness in Greece, Cyprus, and each of the other countries of western Europe, all of which had uninterrupted vital registration time series from pre-1990 to either 2014 or 2015. Spatiotemporal effects and covariates were used to extend estimates to 2016. Under-5 mortality and mortality for ages 15–59 years were estimated separately and linked using empirical life tables to derive age-specific and sex-specific all-cause mortality. The lowest observed risk of death for each age group in total populations of greater than 5 million was summed to construct a global standard life expectancy, which was 86·6 years at birth for GBD 2016.

Cause-specific deaths and years of life lost (YLLs) were estimated for underlying causes of mortality, ensuring that the sum of all specific causes is equal to all-cause mortality. Each death was assigned to a single cause. Vital registration and census death data were adjusted for incompleteness and misclassification (eg, prostate cancer in a female); non-specific and intermediate codes (eg, sepsis, heart failure, unknown causes) were redistributed using age-specific, sex-specific, and geography-specific statistical redistribution methods before modelling. The most commonly used estimation method was with the Cause of Death Ensemble model (CODEm). CODEm uses a train-test-test approach, first testing all combinations of selected country-level covariates and their relation to in-sample data, then ranking those component models based on out-of-sample predictive validity to construct weighted ensembles. All ensemble models are again ranked on a second round of out-of-sample predictive validity to select a final model. Cause-specific fractions were multiplied by all-cause mortality estimates to calculate cause-specific deaths, then scaled with all other causes to match all-cause mortality. YLLs were calculated by multiplying age-specific deaths by global standard life expectancy at age of death.

### Health expenditure

Methods for estimating total health expenditure, government health expenditure, out-of-pocket health expenditure, and prepaid public health expenditure have been described previously.^26^ Briefly, health spending data were extracted for 1995 to 2014 in national currency units and were divided by GDP reported by WHO, then multiplied by GDP per capita in 2015 purchasing-power-parity-adjusted currency.^26^ We added a segmented correlation analysis (Spearman's rank correlation coefficient) per financial period of interest (2000–09; 2010–14) to assess the relation between trends in health expenditure and all-cause mortality.^27^

### Non-fatal health loss as expressed by YLDs

Epidemiological data from systematic literature reviews, health surveys, surveillance systems, disease registries, and hospital and claims databases were used to generate cause-specific and sequela-specific prevalence and incidence estimates using a variety of modelling approaches, of which Bayesian meta-regression compartmental modelling in DisMod-MR 2.1 was the most common.^28^ Disability weights for each unique health state were derived from population surveys of more than 60 000 respondents completed for GBD 2010 and GBD 2013.^29^, ^30^ A microsimulation framework was then used to adjust for comorbidity, and YLDs for each cause were calculated by multiplying prevalence and corresponding disability weights for each sequela of each cause.^23^

### Risk factor estimation and SEVs

The GBD 2016 comparative risk assessment (CRA) framework classified each of 84 risk factors and clusters of risk factors into one of three categories: behavioural, environmental and occupational, or metabolic. Data on risk factor exposure levels were identified, evaluated, and modelled using similar approaches to non-fatal models, with added emphasis on accurately fitting distributions of exposure for continuous and polytomous risk factors. Quantitative relative risk was estimated for each risk-outcome pair, and population attributable fraction statistics were calculated using standard GBD CRA methods.^25^ SEVs represent risk-weighted sums of total exposure in each population, scaled from 0 to 100.

### Uncertainty estimation

Uncertainty for each metric was derived from 1000 draws from the distribution of each estimation step by age, sex, and location for each year included in the GBD 2016 analysis; lower and upper uncertainty intervals (UIs) represent the ordinal 25th and 975th draws of each quantity. UIs allow final estimates to reflect the combined uncertainty of multiple modelling steps. UIs for mortality and YLLs reflect uncertainty in regression coefficients, uncertainty due to sampling and non-sampling error in the cause-of-death data, uncertainty due to various model specifications, and uncertainty in the levels of all-cause mortality. UIs for YLDs reflect uncertainty in prevalence estimates, distribution of severity within each cause, and disability weight valuations. Aggregation of uncertainty across age, sex, and location was done on each draw assuming no correlation.^31^

### Role of the funding source

The funders of the study had no role in the study design, data collection, data analysis, data interpretation, writing of the report, or the decision to submit the article for publication. The authors had access to the data in the study and had final responsibility for the decision to submit for publication.

## Results

### Mortality and causes of death pre-austerity and post-austerity

The all-age, all-cause mortality rate in Greece was 1174·9 (95% UI 1107·4–1243·2) deaths per 100 000 in 2016 compared with 997·8 (975·4–1018) in 2010 and 944·5 (923·1–964·5) in 2000. This finding corresponded to a 0·55% (95% UI·24 to 0·85) ARC from 2000 to 2010, followed by a 2·72% (1·65 to 3·74) annualised increase from 2010 to 2016—a five-times greater ARC post-austerity, with evidence of continuing acceleration (table). The rise in the ARC for all-age mortality was also threefold higher in Greece post-austerity than the 0·86% (95% UI 0·54 to 1·17) rise seen across western Europe for the same period, which was in the opposite direction of the global estimate of a 0·7% (−0·92 to −0·48) fall in all-age mortality from 2010 to 2016. The ARC for all-age mortality in Cyprus increased by 0·65% annually for 2010 to 2016, which was more favourable than regional and global estimates, but was still a retrogression compared with 2000–10 estimates (ARC −1·57% [–1·91 to −1·18]). In terms of age-standardised mortality, Greece's ARC was among the worst-performing countries in western Europe from 2010 to 2016 (appendix), with a reduction from −1·61 (−1·91 to −1·30) from 2000 to 2010 to a marginally negative ARC of −0·87 (−2·03 to 0·20).

**Table:** Mortality estimates for Greece, Cyprus, western Europe, and globally in 2000, 2010, and 2016

	**Number of deaths (all ages)**	**Rate of death (all ages, per 100 000)**	**Rate of death (age-standardised, per 100 000)**
**Greece**			
2000	103 470 (101 126–105 663)	944·5 (923·1 to 964·5)	630·6 (616·4 to 643·8)
2010	111 720 (109 215–113 992)	997·8 (975·4 to 1018)	537·1 (525·1 to 548·1)
2016	127 694 (120 356–135 108)	1174·9 (1107·4 to 1243·2)	509·7 (478·9 to 541·4)
ARC 2000–10	..	0·55% (0·24 to 0·85)	−1·61 (−1·91 to −1·30)
ARC 2010–16	..	2·72% (1·65 to 3·74)	−0·87 (−2·03 to 0·20)
**Cyprus**			
2000	6236 (6059–6427)	893·2 (867·8–920·5)	740·3 (718·8 to 762·8)
2010	6431 (6227–6658)	763·6 (739·5 to 790·7)	566·6 (548·5 to 586·6)
2016	7229 (6919–7566)	793·9 (759·8 to 830·9)	538·1 (514·6 to 563·8)
ARC 2000–10	..	−1·57% (−1·91 to −1·18)	−2·67 (−3·03 to −2·29)
ARC 2010–16	..	0·65% (0·13 to 1·14)	−0·86 (−1·4 to −0·36)
**Western Europe**			
2000	3 840 157 (3 803 360–3 875 763)	971 (961·7 to 980·0)	624·6 (618·5 to 630·5)
2010	3 876 317 (3 837 211–3 913 200)	929 (919·6 to 937·8)	508·7 (503·4 to 513·8)
2016	4 189 406 (4 104 536–4 272 881)	978·3 (958·5 to 997·8)	475·1 (465·3 to 484·9)
ARC 2000–10	..	−0·44% (−0·57 to −0·3)	−2·05 (−2·18 to −1·91)
ARC 2010–16	..	0·86% (0·54 to 1·17)	−1·14 (−1·48 to −0·81)
**Global**			
2000	51 464 936 (51 060 928–51 865 787)	842·2 (835·6 to 848·8)	1111·3 (1103·3 to 1119·5)
2010	53 304 029 (52 825 670–53 789 494)	771·6 (764·6 to 778·6)	928 (920·3 to 935·7)
2016	54 698 580 (54 028 682–55 514 892)	739·9 (730·9 to 751·0)	832·7 (822·7 to 845·0)
ARC 2000–10	..	−0·88% (−0·98 to −0·78)	−1·80% (−1·89 to −1·71)
ARC 2010–16	..	−0·70% (−0·92 to −0·48)	−1·81% (−2·02 to −1·59)

Ranges are smallest to highest. ARC=annualised rate of change.

**Figure 1:**
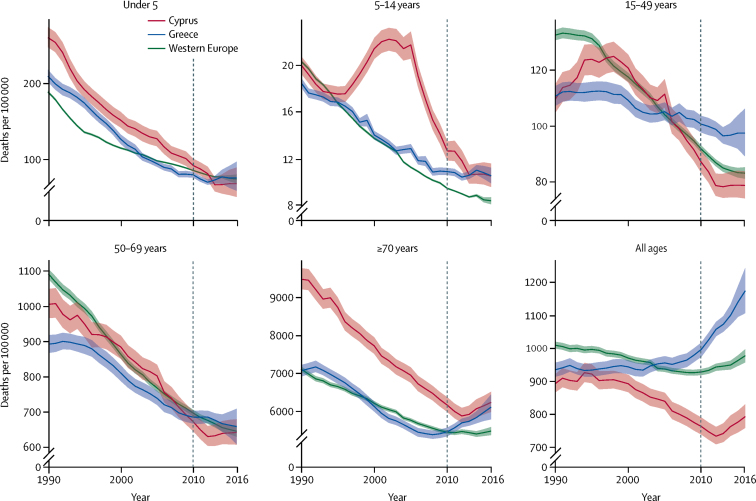
All-cause mortality by age group from 1990–2016 in Greece, Cyprus, and western Europe

Mortality trends in Greece were especially unfavourable in adults 15 years or older, with the largest increases seen in those aged 70 years or older (figure 1). Disaggregation of trends in age-specific mortality revealed that, although Greece had slower improvement than Cyprus and western Europe in most age groups from 2000 to 2010, the ARC of age-specific all-cause mortality was similar across all three locations in ages 15 and older from 2010 to 2016, albeit worse from birth to age 14 in Greece (appendix). In parallel, there was evidence of a rapid change in population structure from 2010 to 2016, with a reduction of population in age groups of 15–34 years and increases in population age groups of 55–59 years and 75 years and older (appendix). In comparison with Greece's ageing population, all population age groups over 20 years of age in Cyprus recorded slight increases during the same period (appendix).

**Figure 2:**
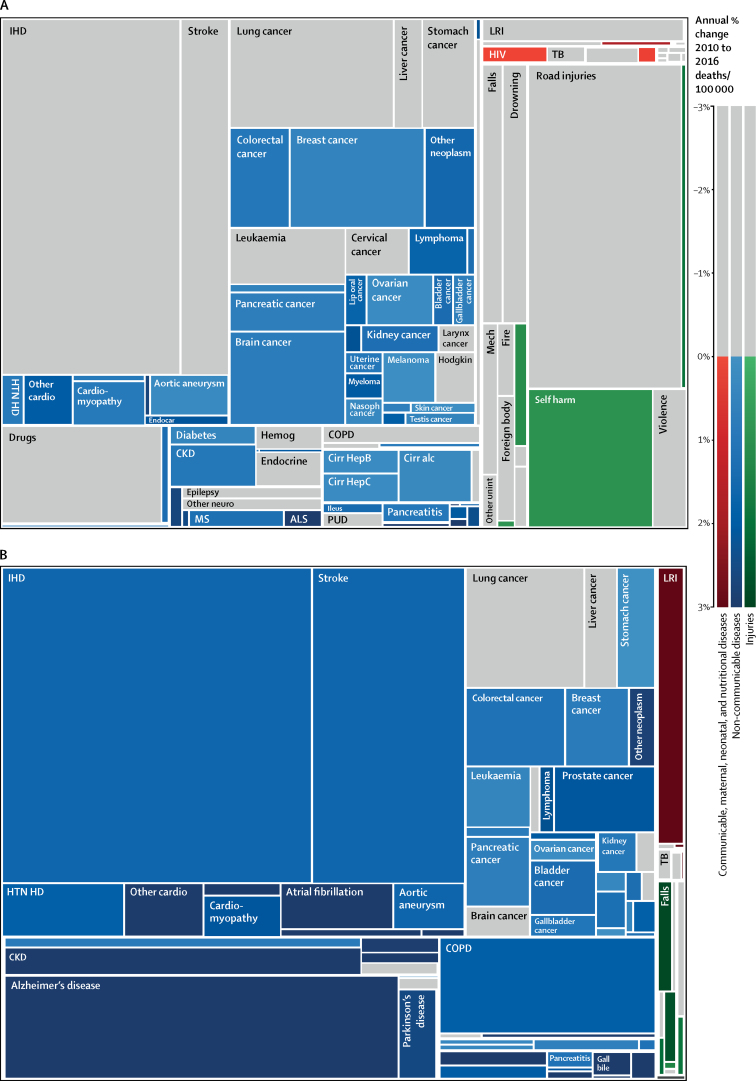
Proportional distribution of causes of death for age 15–49 years (A) and ≥70 years (B) in Greece Figure shows all level 3 Global Burden of Diseases, Injuries, and Risk Factors causes of death with colouring of causes that have increased from 2010 to 2016, as deaths per 100 000. IHD=ischaemic heart disease. LRI=lower respiratory infection. TB=tuberculosis. Mech=exposure to mechanical forces. Fire=fire injuries. Hodgkin=Hodgkin's lymphoma. HTN HD=hypertensive heart disease. Other cardio=other cardiovascular disease. Endocar=endocarditis. Nasoph=nasopharyngeal disease. Hemog=haemoglobinopathies and haemolytic anemias. COPD=chronic obstructive pulmonary disease. CKD=chronic kidney disease. Cirr=cirrhosis. Other neuro=other neurological disease. MS=multiple sclerosis. ALS=amyotrophic lateral sclerosis. PUD=peptic ulcer disease. Other unint=other unintentional injuries. Gall bile= gallbladder and biliary diseases.

As for specific causes of death, adverse effects of medical treatment, self-harm, and several types of cancer stood out as consistently increasing in Greece across all ages (figure 2). Within specific age groups, other causes are apparent, with rapid increases in deaths due to neonatal haemolytic disease and neonatal sepsis in children younger than 5 years, and prominent increases in self-harm among adolescents and young adults. Greek adults aged 15–49 years had increased mortality due to HIV, several treatable neoplasms, all types of cirrhosis, neurological disorders (eg, multiple sclerosis, motor neuron disease), chronic kidney disease, and most types of cardiovascular disease except for ischaemic heart disease and stroke (appendix). This result contrasts with Cyprus, where drug use was the only top ten cause of death that increased in the 15–49 years age groups, and with western Europe, where no causes increased from 2010 to 2016. In adults aged 70 years or older, only a subset of causes of death increased in Cyprus and western Europe, but nearly all increased—and increased more rapidly—in Greece between 2010 and 2016 (appendix).

### Health expenditure

**Figure 3:**
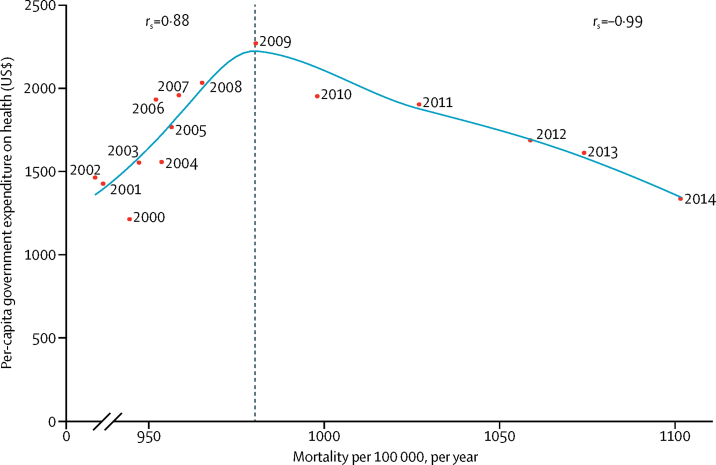
Mortality according to total health expenditure in Greece

Total health expenditure in Greece increased consistently from 2000 to 2008 (the year Greece's austerity programme commenced), at which point total health expenditure was cut by US$130·32 per capita and has since remained in decline (figure 3). When assessing the associations between mortality rates and total health expenditure in Greece, two distinct patterns were observed: during the 2000–08 period, an interrupted increase in total health expenditure was associated with a slow incremental increase in total mortality (Spearman's *r*_s_=0·88), whereas from 2009 to 2014, this relationship was reversed (Spearman's *r*_s_*=*–0·99) as the rate of increase in all-cause mortality hastened in the setting of compound decreases in total health expenditure. The same trend was noted for total health expenditure in Cyprus, but the cuts were not as substantial and there was a 1-year lag compared with Greece. Associations between total health expenditure and mortality were also different in Cyprus (appendix), because all-cause death rates decreased continuously until 2012. Examining more granular components of total health expenditure, including government, out-of-pocket, and prepaid public health expenditure revealed broadly similar trends (appendix). Prepaid public health expenditure dropped by 14% from 2008 to 2009, consistent with the onset of austerity measures, but increased to pre-austerity levels thereafter, which could be consistent with the pursuit of private health care due to inefficiencies of the public health system (appendix).

### YLDs pre-austerity and post-austerity

Trends in non-fatal health loss were less dramatic than those of mortality. A consistent pattern for causes of YLDs was noted across Greece, Cyprus, and all of western Europe. Low-back and neck pain, migraine, depressive disorders, anxiety disorders, and skin diseases were the top five causes of YLDs in all areas for both 2000 and 2016, whereas oral disorders (caries of permanent teeth and chronic periodontal disease) and tension-type headache were the most prevalent conditions (appendix). HIV increased in both Cyprus and Greece from 2000 to 2016 in adults.

### Risk factors

Age-standardised SEVs for each of the most detailed risk factors, which represent risk-weighted sums of total exposure in 2000, 2010, and 2016 show that Greece has higher exposure to several risks than either Cyprus or western Europe, including smoking, ambient ozone pollution, high body-mass index (BMI), and diet low in omega-3 and polyunsaturated fatty acids. Behavioural and metabolic risk factors accounted for the majority of attributable YLDs in Greece and Cyprus in 2016. The appendix shows the trends in age-standardised YLDs due to these factors from 2000 to 2016. In western Europe, 936·2 (663·2–1283·9) YLDs per 100 000 were attributable to metabolic risks in 2016, which was a slight decrease compared with 2010. The corresponding 2016 estimates for Greece and Cyprus were 892·1 (632·3–1229·2) and 1055·2 (749·7–1436·4) per 100 000 YLDs attributable to metabolic risks, respectively. The burden attributable to behavioural risks recorded a steady fall for western Europe and Cyprus from 2000 to 2016, whereas an opposite trend was observed in Greece, with an overall increase of 2% in YLDs attributable to behavioural risks from 2000 to 2016. Disaggregation of metabolic risks revealed an increase in YLDs attributable mainly to high BMI, high fasting plasma glucose, and high blood pressure in Greece since 2000. The risk attributable to tobacco continuously increased in Greece since 2000, ranking first among behavioural risks with a 2·25% increase in YLD percentage attributable to tobacco from 2010 to 2016. Alcohol consumption and dietary risks ranked second and third, respectively. By contrast, during the same period, YLDs attributable to tobacco reduced by 8·1% in Cyprus and 13·2% in western Europe.

**Figure 4:**
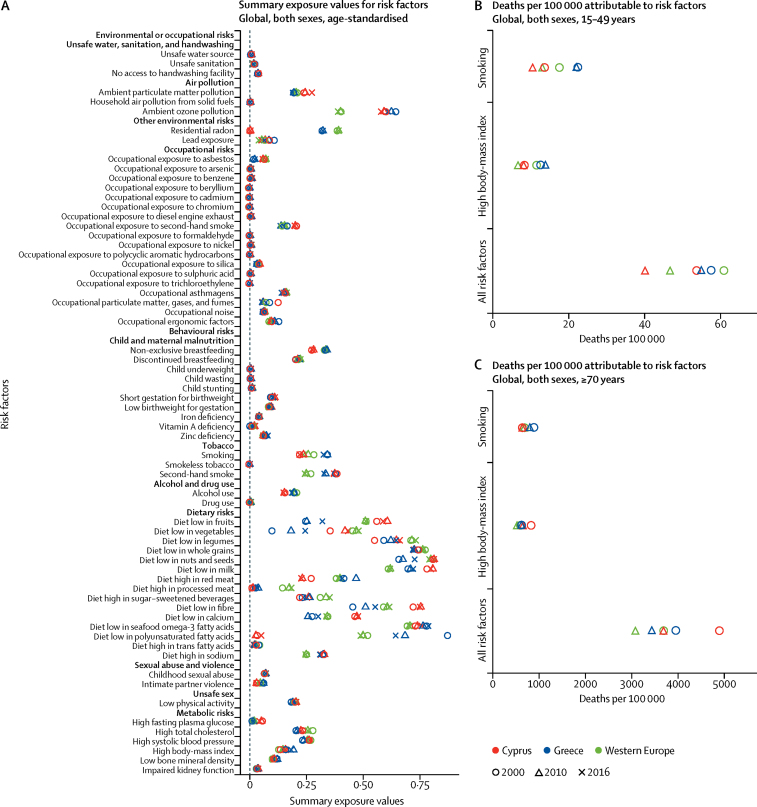
Risk factor analysis of mortality in Greece (A) Summary exposure values. (B) Deaths per 100 000 attributable to risk factors age 15–49 years. (C) Deaths per 100 000 attributable to risk factors age ≥70 years.

When paired with age-specific mortality rates to calculate smoking-attributable mortality, approximately 70% of the 2016 gap in all-cause mortality between Greece and western Europe for ages 15–49 years could be attributed to excess smoking in Greece, whereas much of the remainder can be attributed to high BMI. For those aged 70 years or older, however, only 25% of the excess mortality in Greece could be attributed to smoking and approximately 15% to high BMI (figure 4).

## Discussion

All-age mortality in Greece was stable from 1990 to 2000, began to increase from 2000 to 2010, and accelerated upward from 2010 to 2016. The inflection point around 2010 was most pronounced in the very young and very old and, on aggregate, led to a 50% slowdown in the reduction in age-standardised mortality in Greece since 2010. Much of the increase in all-age mortality was concentrated in adults. Reasons for the increase included population ageing, a net fall in the number of young adults living in Greece, and a higher-than-average exposure to behavioural and metabolic risk factors. Many of the causes of death that increased in Greece are potentially responsive to care, including HIV, neoplasms, cirrhosis, neurological disorders, chronic kidney disease, and most types of cardiovascular disease. Improvements in child mortality in Greece have also stagnated since 2000, with increases in deaths due to neonatal haemolytic disease and neonatal sepsis since 2010, both of which might also reflect reduced health system performance.

Findings of reduced improvement in age-standardised mortality after austerity are consistent with those of Laliotis and colleagues,^9^ who also reported slowing of overall mortality reduction in Greece after the financial crisis despite differences in data sources and methods (official statistics from Hellenic Statutory Authority and EUROSTAT *vs* vital registration adjusted for incompleteness, misclassification, and comprehensive statistical modelling). Laliotis and colleagues also noted that those older than age 75 years had more negative effects than remaining adult age groups, identified a reversed epidemiological transition manifested as a nearly 10% increase in mortality due to communicable, maternal, neonatal, and nutritional diseases, and reported a worsening of mental health in Greece. The GBD 2016 findings of static child mortality also align with reports of increases in infant mortality and stillbirths post-austerity.^32^

Several plausible explanations could account for the trends of disease burden observed in Greece recently; the full explanation is probably multifactorial. First, population ageing in Greece preceded the economic crisis and could have contributed to the slow but measurable increase in all-cause mortality rates since 2000. Acceleration of population ageing since 2010 could be due to the massive emigration of early to mid-career educated professionals in pursuit of financial stability, in what has been referred to as brain drain.^33^ Our findings for cause-specific mortality after 2010 do not support ageing as the only culprit, however, because increases were also noted in deaths due to neonatal haemolytic disease and neonatal sepsis in children younger than 5 years, self-harm among adolescents and young adults, HIV in young adults, and several treatable cancers in younger adults. At the same time, the demographic transition in Greece cannot be considered independent to the economic crisis, because emigration can be triggered by crisis-related increments in unemployment, reductions in wages, and stringent taxation schedules.^33^

Second, reversal in per-capita health expenditure in Greece in 2009–10, despite a continually ageing population that would have been expected to lead to increased spending, might have caused a fiscal and organisational shock to the health-care system. Since the implementation of the austerity programme, Greece has reduced its ratio of health-care expenditure to GDP to one of the lowest within the EU, with 50% less public hospital funding in 2015 than in 2009.^34^ This reduction has left hospitals with a deficit in basic supplies, while consumers are challenged by transient drug shortages.^35^ Concurrently, nearly a quarter of the population lost health insurance from the national health-care programme due to longstanding unemployment, while more than 20% reductions in the minimum wage reduced consumer buying power.^34^ Uncoupling of causes of health loss due to existing unhealthy behaviours and rooted inefficiencies of the health system from those due to the effects of austerity-related health policies is challenging and limited by data availability. Nonetheless, steep changes in health loss indicators since 2010 support a role of the austerity measures in accelerating the pre-existing health burden since 2000. Without careful mitigation and planning within the health-care system, large cuts could challenge the ability to maintain services at a level required to meet the growing health needs of an ageing population.^17^, ^36^, ^37^ Constriction of health-care provision in Greece has been associated with a decrement in self-rated health following the onset of the austerity programme.^13^, ^38^, ^39^ The austerity measures were coupled with societal measures and structural reforms to ameliorate any adverse effects to the population's wellbeing, but these reforms have not yet yielded a measurable effect on health burden.^39^

Third, elevated BMI, smoking, and unhealthy diet are increased in Greece, which is responsible for much of the excess mortality in adults aged 15–49 years compared with Cyprus and western Europe. However, age-standardised mortality attributable to tobacco fell more quickly after austerity than before (−1·21% *vs* −1·36% annually), as did high BMI (−0·75% *vs* −1·52% annually), whereas the age-standardised mortality attributable to unhealthy dietary habits increased (−1·86% *vs* −0·88% annually). Although increasing trends in risk factors might be responsible for much of the excess mortality in younger adults, this study found that risk factors account for only a minority of the excess mortality in older adults. Furthermore, elevated BMI, smoking, and unhealthy diets could be linked to deficiencies in the establishment or implementation of health policies and social inertia, as well as direct and indirect effects of the economic crisis.^3^ Behavioural health policies encompassing health and nutrition education in Greece need improvement, with recent reports indicating the need for enhancement of nutritional services, reduction of disparities in health-care access, and expansion of limited rural primary care services^40^ to improve regional performance of areas such as the Aegean islands.^41^ Notably, the number of individuals with unmet health-care needs nearly doubled since 2010, with a considerable fraction reporting health-care cost as the main reason for not receiving the recommended health-care services.^17^ These reports point to a potential additive effect of the economic crisis to existing deficiencies in health services.

Fourth, and relatedly, risk factors associated with disease burden are also linked to region-specific inertias and could therefore extend beyond the structural deficiencies of the Greek health-care system. Greece has adopted the EU recommendation for smoke-free laws by setting one of the most stringent smoke-free policies among all EU countries, which called for completely banning smoking in enclosed public spaces.^42^ The implementation, however, has been largely unsuccessful.^43^, ^44^ Interestingly, an analysis of the Hellas Health survey^44^ reported a 5% reduction in the prevalence of smokers in Greece from 2008 to 2011. This reduction might be a joint result of a tobacco taxation programme and reduced spending power.^44^ The lack of a major effect of the crisis on health loss due to cardiovascular diseases, as reported by many investigators,^17^, ^44^ might be explained by lifestyle changes related to the financial crisis, such as reduction in tobacco usage following the increased taxation programme and increase of physical activity related to reduced usage of private transportation vehicles.^17^

Fifth, contemporaneous to the economic crisis, Greece has been hosting an increasing number of refugees from Syria notably, which could pose an additional challenge for national health and welfare systems and warrants new investigations on the effect on the national health status.

Similar to the slower reduction in health loss noted in Greece, Cypriot estimates showed that overall fatal health measures continued to decrease at a similar slower pace in the post-austerity era compared with 2000–10. ARC in age-standardised mortality fell at −0·86% post-austerity compared with −2·67% pre-austerity. Although Cyprus also implemented an austerity programme, the effect of the crisis on the health of residents of Cyprus as compared with those in Greece is consistent with the development of the economic crisis in each country. The Greek financial crisis was handled by an ongoing austerity programme based on taxation and broad cuts that still has not stabilised the economy or produced sustainable control of the deficit.^38^ By comparison, the austerity programme imposed in Cyprus in 2013 entailed an aggressive cut,^45^ that led to transient turbulence but was followed by a programme focused on growth and structural reform of the public sector, including the health-care system, which led to an early exit from the austerity programme for Cyprus.^46^

The trajectories of health outcomes in Greece and Cyprus in the post-austerity era offer valuable lessons for policy makers aiming to limit health effects in regions contemplating broad-based cuts. In Cyprus, austerity measures had direct effects on health care that aimed to increase the efficiency and sustainability of the national health-care system.^46^ The main health-care reform measures taken concerned establishing user charges (ie, co-payments), rationalising diagnostic examinations, and introducing guidelines to minimise waste of health resources.^47^ In Greece, austerity measures aimed directly at the reduction of the health-care expenditure to GDP ratio without a structured plan for reform.^48^ Notably, Greece holds the second-highest rank among EU countries for the highest number of doctors per 1000 inhabitants, indicating the availability of a medical workforce that is not efficiently utilised.^49^ In addition to Greece and Cyprus, other European countries including Spain, Portugal, Italy, and Ireland have been faced with austerity measures or received bailout packages that affected their health-care systems. There were varying effects on population health among these countries. In Spain, mortality dropped during the economic crisis, whereas in Ireland male suicide rates increased by more than 50% following the onset of the crisis.^14^ The present analysis revealed that, when comparing age-standardised mortality rates among these countries that were affected by the regional financial crisis, there were varying trends for overall worse outcomes, which were more pronounced for Cyprus, Portugal, and Greece.

The present analysis shares the limitations of the GBD 2016 study, first and foremost the challenges of capturing all sources of uncertainty, lags in data availability, variation in coding practices and other biases, and limitations of existing analytical tools, which might not fully capture temporal trends in mortality, incidence, and prevalence. Second, and relatedly, these analyses are based on comparatively sparse data from more recent years, especially for non-fatal health outcomes. Third, lack of adequately matched information on health-care fund allocation by age and condition precluded analysis of its association with estimated health outcomes. It cannot be excluded that changes in population structure or indirect effects of the economic crisis and not the austerity-related reduction of GDP allocation to health care were the main drives of health loss in Greece. Fourth, although Cyprus and Greece share language and socioeconomic structures in general, other differences between them could bias our findings.

In conclusion, there is evidence of a disproportionate decrement in the health of Greeks compared with regional populations, which parallels the course of the economic crisis. From 2010 to 2016, Greece was faced with a five-times greater rate of annual all-cause mortality increase and a more modest increase in non-fatal health loss compared with pre-austerity. During the same period the ARC of age-standardised mortality reduced to nearly half the ARC for the 2000 to 2010 period. The findings of steep quantitative changes in mortality trends and qualitative changes in mortality causes with a rise in communicable, maternal, neonatal, and nutritional diseases since 2010 suggest that an effect of the abruptly reduced government health expenditure on population health is likely. Nonetheless, the marginally negative ARC of age-standardised mortality reflects notable changes in population age structure in Greece when compared with the crude estimates for total mortality, probably related to emigration of young adults. There is a need for targeted studies to disaggregate the causes of accelerated population ageing and to determine the needs of the health-care system to cope with these changes in population structure. The findings of increases in total deaths and accelerated population ageing call for specific focus from health policy makers to determine the public health needs to cope with these changes.

Correspondence to: Dr Georgios A Kotsakis, University of Washington, School of Dentistry, Seattle, WA 98195-7444, USA **kotsakis@uw.edu**

For more on the **GATHER statement** see http://gatherstatement.org/
