# Supplementary material for: The burden of disease in Greece, health loss, risk factors, and health financing, 2000–16: an analysis of the Global Burden of Disease Study 2016
Source: Lancet Public Health. 2018 Jul 25;3(8):e395–406. doi: 10.1016/S2468-2667(18)30130-0 (PMC6079016; doi:10.1016/S2468-2667(18)30130-0)

THE LANCET
Public Health

Supplementary appendix

This appendix formed part of the original submission and has been peer reviewed. We post it as supplied by the authors.

Supplement to: Global Burden of Disease 2016 Greece Collaborators. The burden of disease in Greece, health loss, risk factors, and health financing, 2000–16: an analysis of the Global Burden of Disease Study 2016. *Lancet Public Health* 2018; published online July 25. http://dx.doi.org/10.1016/S2468-2667(18)30130-0.

Supplemental Results Appendix to The burden of disease in Greece in a time of crisis

Table of Contents

Appendix Figure 1. Individual trajectories of age-standardised mortality are depicted from Western European countries that have been affected by the financial crisis to provide further comparisons to estimated health loss in Greece. 2

Appendix Figure 2. Population structure for Greece and Cyprus in the period 2000–2016. 2

Appendix Figure 3: Annualised rate of change in age standardised all-cause mortality, 2000-2010 and 2010-2016, by GBD age group in Greece, Cyprus and Western Europe for both sexes........ 3

Appendix Figure 4. Distribution of causes of death for 15 to 49 years (Panels A-C) and 70+ years (Panels D-F) in Greece (Panels A and D), Cyprus (Panels B and E), and Western Europe (Panels C and F) with highlighting of causes that have increased from 2010 to 2016. 4

Appendix Figure 5a. Top 20 causes of death in Greece, Cyprus, and Western Europe, age-standardized with mean percent change........ 5

Appendix Figure 5b. Top 20 causes of death in Greece, Cyprus, and Western Europe, age groups 15-49 and 70+ with mean percent change........ 6

Appendix Figure 6. Health expenditure compared to mortality in Greece and Cyprus 8

Cyprus Government health expenditure (GHE), Out of pocket health expenditure (OOP), Prepaid public health expenditure (PPP), and total health expenditure (THE) from 2000 to 2014 compared to mortality rate (all ages) and age-standardised mortality rate........ 8

Greece Government health expenditure (GHE), Out of pocket health expenditure (OOP), Prepaid public health expenditure (PPP), and total health expenditure (THE) from 2000 to 2014 compared to mortality rate (all ages) and age-standardised mortality rate........ 9

Appendix Figure 7. Top-ranked causes of years lived with disability (YLDs) at all ages and both sexes in Greece, Cyprus, and Western Europe for 2000 and 2016 10

Appendix Figure 8. YLDs per 100,000 attributable to metabolic and behavioural risk factors in Greece, Cyprus, Western Europe, and globally, 2000–2016........ 11

Appendix Figure 1. Individual trajectories of age-standardised mortality are depicted from Western European countries that have been affected by the financial crisis to provide further comparisons to estimated health loss in Greece.

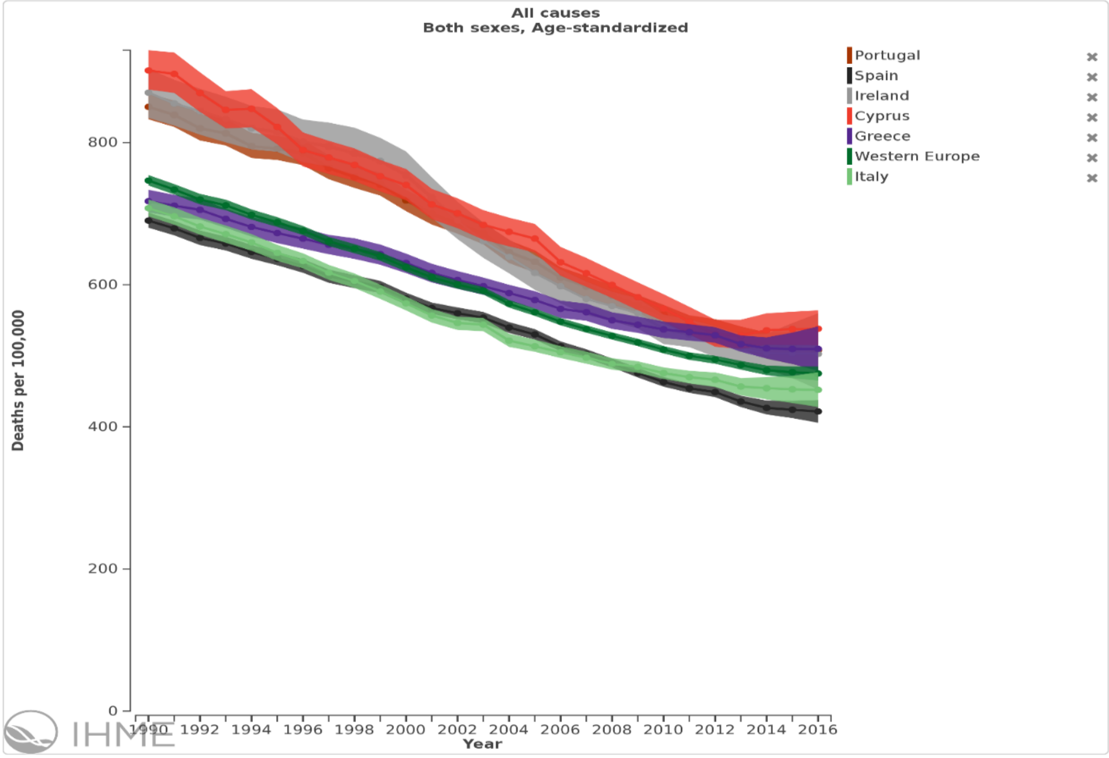

Appendix Figure 2. Population structure for Greece and Cyprus in the period 2000–2016.

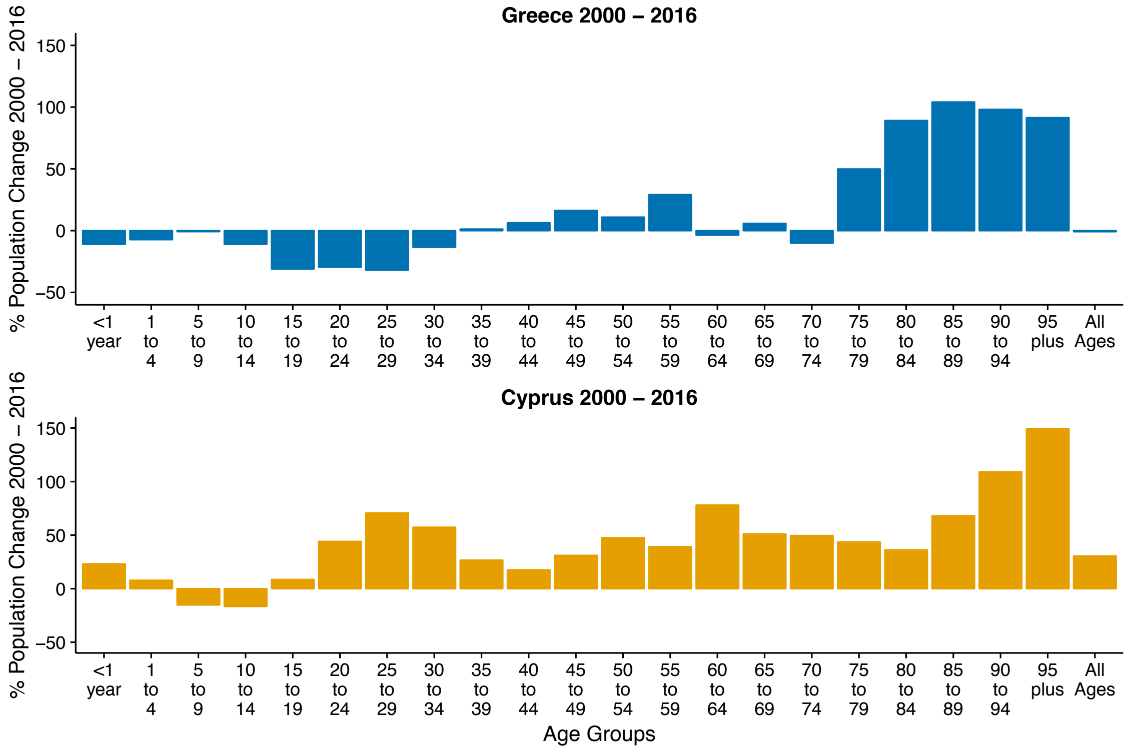

Appendix Figure 3: Annualised rate of change in age standardised all-cause mortality, 2000-2010 and 2010-2016, by GBD age group in Greece, Cyprus and Western Europe for both sexes.

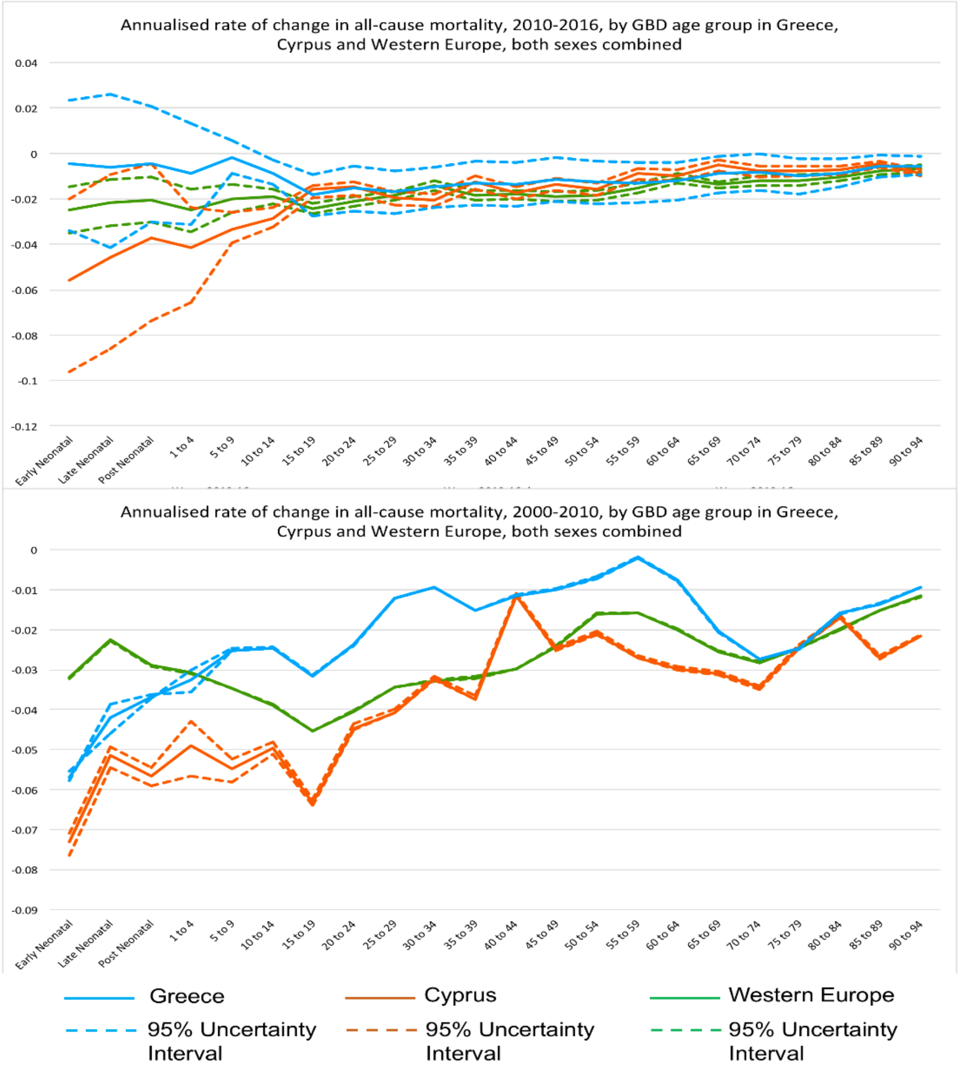

Appendix Figure 4. Distribution of causes of death for 15 to 49 years (Panels A-C) and 70+ years (Panels D-F) in Greece (Panels A and D), Cyprus (Panels B and E), and Western Europe (Panels C and F) with highlighting of causes that have increased from 2010 to 2016.

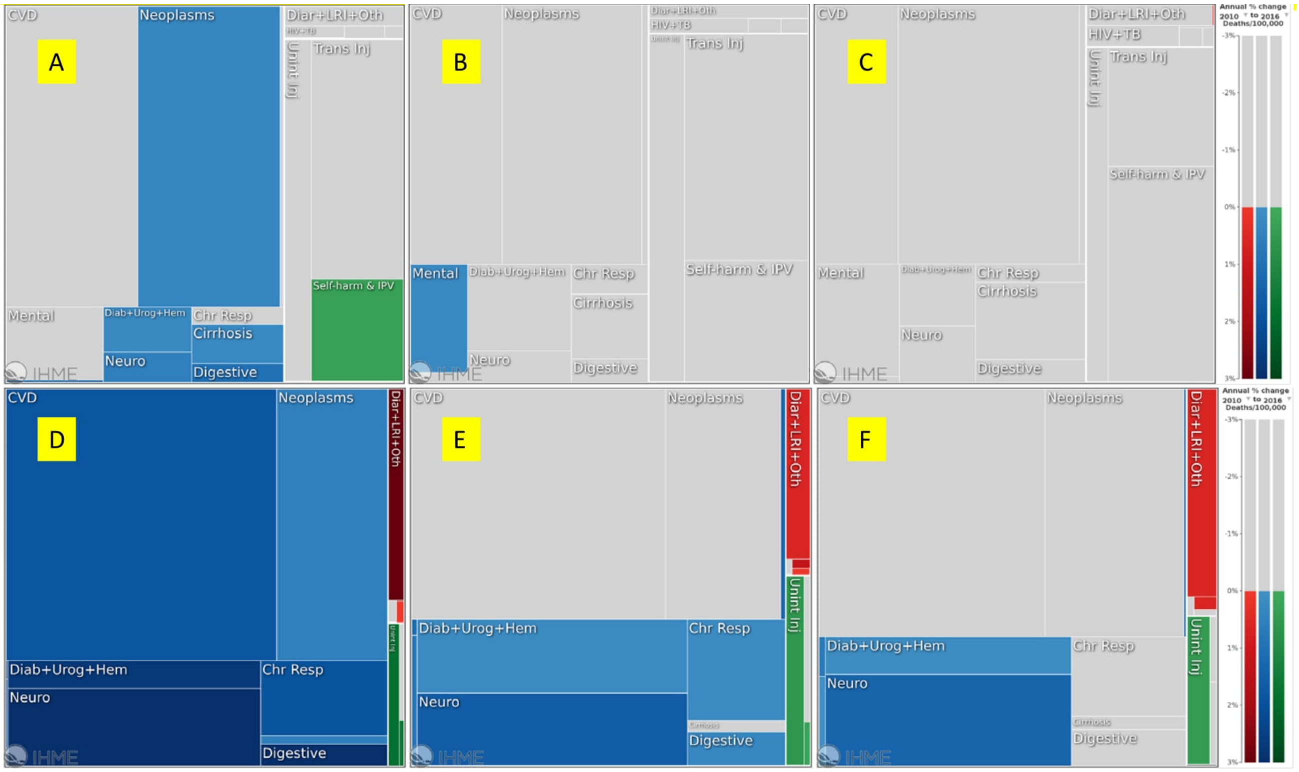

Appendix Figure 5a. Top 20 causes of death in Greece, Cyprus, and Western Europe, age-standardized with mean percent change

Top 20 causes of Deaths in Cyprus, Age-Standardized, with mean perent change

Leading causes 2000	Leading causes 2010	Percent Change (rates) 2000 - 2010	Leading causes 2016	Percent Change (rates) 2010 - 2016
1 Ischemic heart disease	1 Ischemic heart disease	-34.8% (-39 to -30%)	1 Ischemic heart disease	-8.3% (-12.3 to -4%)
2 Stroke	2 Alzheimer disease	-6.7% (-11.6 to -0.8%)	2 Alzheimer disease	0.5% (-3 to 4.1%)
3 Diabetes	3 Stroke	-38.1% (-44.1 to -30.9%)	3 Stroke	-9.1% (-15.5 to -2.4%)
4 Alzheimer disease	4 Diabetes	-28.3% (-35 to -20.4%)	4 Diabetes	-7.8% (-14.7 to -1.2%)
5 COPD	5 Lung cancer	22.1% (9.7 to 37.1%)	5 Lung cancer	-6.8% (-13.4 to 0.2%)
6 Road injuries	6 COPD	-25.1% (-31.5 to -17.9%)	6 COPD	-4.9% (-11.3 to 1.5%)
7 Chronic kidney disease	7 Chronic kidney disease	-18.6% (-26.4 to -10.5%)	7 Chronic kidney disease	0.5% (-6.4 to 8%)
8 Lung cancer	8 Colorectal cancer	4.3% (-6.9 to 17%)	8 Colorectal cancer	-2.3% (-8.9 to 4.7%)
9 Lower respiratory infect	9 Road injuries	-44.6% (-48.6 to -39%)	9 Other cardiovascular	-3.4% (-12.2 to 5.4%)
10 Other cardiovascular	10 Other cardiovascular	-21.4% (-31.3 to -9.9%)	10 Breast cancer	-2.7% (-10.8 to 5.4%)
11 Breast cancer	11 Breast cancer	-4% (-27 to 11.9%)	11 Road injuries	-14.8% (-20.3 to -9.1%)
12 Colorectal cancer	12 Lower respiratory infect	-34.1% (-42.1 to -26.1%)	12 Lower respiratory infect	-5.8% (-15.1 to 2.9%)
13 Hypertensive heart disease	13 Prostate cancer	-4.4% (-24.4 to 11.7%)	13 Prostate cancer	-1% (-11.1 to 8.3%)
14 Prostate cancer	14 Hypertensive heart disease	-25% (-36.8 to -7.6%)	14 Hypertensive heart disease	-4.3% (-13.5 to 4.8%)
15 Falls	15 Pancreatic cancer	28.6% (17.4 to 41.4%)	15 Pancreatic cancer	-0.5% (-7.6 to 6.9%)
16 Stomach cancer	16 Stomach cancer	-1.8% (-9.9 to 7.3%)	16 Stomach cancer	-5.1% (-11.4 to 1%)
17 Aortic aneurysm	17 Leukemia	0.6% (-26.2 to 16.4%)	17 Leukemia	-1.9% (-7.6 to 5.3%)
18 Leukemia	18 Falls	-21.1% (-28.5 to -13%)	18 Falls	-1.6% (-8.3 to 5.7%)
19 Urinary diseases	19 Other neoplasms	29.8% (-6.4 to 47.3%)	19 Other neoplasms	2.4% (-4.4 to 10.7%)
20 Atrial fibrillation	20 Urinary diseases	-13.2% (-23.3 to -0.6%)	20 Urinary diseases	2% (-6.2 to 11.7%)
24 Pancreatic cancer	22 Aortic aneurysm		22 Atrial fibrillation	
30 Other neoplasms	23 Atrial fibrillation		23 Aortic aneurysm	

Top 20 causes of Deaths in Greece, Age-Standardized, with mean perent change

Leading causes 2000	Leading causes 2010	Percent Change (rates) 2000 - 2010	Leading causes 2016	Percent Change (rates) 2010 - 2016
1 Ischemic heart disease	1 Ischemic heart disease	-14.2% (-17.8 to -10%)	1 Ischemic heart disease	-8.2% (-14.5 to -1.6%)
2 Stroke	2 Stroke	-36.9% (-40.8 to -32.9%)	2 Stroke	-7.4% (-14.7 to -0.3%)
3 Alzheimer disease	3 Alzheimer disease	-2.5% (-7.3 to 2.5%)	3 Alzheimer disease	-1% (-7 to 4.9%)
4 Lung cancer	4 Lung cancer	-7.6% (-13.5 to -1.2%)	4 Lung cancer	-5.9% (-14.6 to 3.8%)
5 COPD	5 COPD	35.1% (26.6 to 44.1%)	5 COPD	-7.9% (-14.9 to -0.2%)
6 Road injuries	6 Colorectal cancer	-5.6% (-11.8 to 1%)	6 Colorectal cancer	-3% (-12.4 to 6.7%)
7 Chronic kidney disease	7 Road injuries	-29.2% (-33.2 to -25%)	7 Chronic kidney disease	3.1% (-5.3 to 11.9%)
8 Colorectal cancer	8 Chronic kidney disease	-28.4% (-32.9 to -23.5%)	8 Road injuries	-13% (-21.5 to -3.8%)
9 Lower respiratory infect	9 Breast cancer	-6.4% (-13.7 to 2.5%)	9 Breast cancer	-4.5% (-15.2 to 7.7%)
10 Breast cancer	10 Lower respiratory infect	-26.8% (-33.3 to -19.4%)	10 Lower respiratory infect	0.5% (-9.8 to 11.4%)
11 Liver cancer	11 Stomach cancer	-18.6% (-23.8 to -13.1%)	11 Pancreatic cancer	-2% (-10.7 to 7.4%)
12 Stomach cancer	12 Pancreatic cancer	3.6% (-3.4 to 11%)	12 Stomach cancer	-8.1% (-16.2 to 0.6%)
13 Other cardiovascular	13 Liver cancer	-27.4% (-32.9 to -21.2%)	13 Prostate cancer	-1% (-12.6 to 11.8%)
14 Prostate cancer	14 Prostate cancer	-14% (-23.5 to -0.8%)	14 Hypertensive heart disease	-7.9% (-17.1 to 4.8%)
15 Pancreatic cancer	15 Hypertensive heart disease	65.7% (-14 to 120.3%)	15 Leukemia	-5% (-13.1 to 4%)
16 Leukemia	16 Leukemia	-8.4% (-14.4 to -1.9%)	16 Liver cancer	-15.5% (-23.3 to -7.5%)
17 Brain cancer	17 Brain cancer	-0.4% (-8.3 to 6.1%)	17 Brain cancer	-2.1% (-11.2 to 7.1%)
18 Bladder cancer	18 Diabetes	-6.5% (-13.7 to 0.9%)	18 Other cardiovascular	14.3% (3.8 to 25.8%)
19 Diabetes	19 Bladder cancer	-13.7% (-21.1 to -5.6%)	19 Bladder cancer	-1.6% (-12 to 9.6%)
20 Aortic aneurysm	20 Aortic aneurysm	-7.1% (-14.5 to 1.2%)	20 Diabetes	-3.6% (-12.2 to 5.8%)
24 Hypertensive heart disease	22 Other cardiovascular		21 Aortic aneurysm	

Top 20 causes of Deaths in Western Europe, Age-Standardized, with mean perent change

Leading causes 2000	Leading causes 2010	Percent Change (rates) 2000 - 2010	Leading causes 2016	Percent Change (rates) 2010 - 2016
1 Ischemic heart disease	1 Ischemic heart disease	-34.6% (-36 to -32.9%)	1 Ischemic heart disease	-10.3% (-12.7 to -7.9%)
2 Stroke	2 Alzheimer disease	-4.9% (-7.4 to -2.6%)	2 Alzheimer disease	-1% (-3 to 0.9%)
3 Alzheimer disease	3 Stroke	-32.3% (-34.3 to -30.2%)	3 Stroke	-10.8% (-13.2 to -8.3%)
4 Lung cancer	4 Lung cancer	-7.8% (-10.1 to -5.4%)	4 Lung cancer	-7.9% (-10.7 to -5%)
5 COPD	5 COPD	-15% (-17.3 to -12%)	5 COPD	-6.4% (-9 to -4.1%)
6 Colorectal cancer	6 Colorectal cancer	-12.8% (-15.2 to -10.3%)	6 Colorectal cancer	-6.3% (-10.1 to -2%)
7 Lower respiratory infect	7 Lower respiratory infect	-22.1% (-24.7 to -19.4%)	7 Lower respiratory infect	-5.1% (-8.6 to -1.7%)
8 Breast cancer	8 Breast cancer	-16% (-18.9 to -13.3%)	8 Breast cancer	-8.7% (-12.7 to -4.8%)
9 Other cardiovascular	9 Other cardiovascular	-16.5% (-19.1 to -13.8%)	9 Other cardiovascular	-5.5% (-9.1 to -1.5%)
10 Diabetes	10 Diabetes	-19.4% (-22.2 to -16.5%)	10 Diabetes	-8.8% (-12 to -5.4%)
11 Self-harm	11 Self-harm	-15.3% (-21.5 to -8.5%)	11 Self-harm	-5.6% (-11 to -0.9%)
12 Road injuries	12 Pancreatic cancer	6.2% (2.8 to 9.4%)	12 Pancreatic cancer	-2.6% (-6.5 to 0.9%)
13 Stomach cancer	13 Chronic kidney disease	9.6% (6.4 to 12.9%)	13 Chronic kidney disease	-2.8% (-5.9 to 0.5%)
14 Prostate cancer	14 Prostate cancer	-11.1% (-15.4 to 4.7%)	14 Prostate cancer	0.3% (-4.8 to 5.2%)
15 Pancreatic cancer	15 Stomach cancer	-26% (-27.8 to -24.2%)	15 Stomach cancer	-9.3% (-12.3 to -6.2%)
16 Chronic kidney disease	16 Hypertensive heart disease	0.5% (-21 to 11.8%)	16 Hypertensive heart disease	-5.7% (-10.8 to 0.1%)
17 Cardiomyopathy	17 Falls	-9.2% (-19.2 to -5.3%)	17 Falls	-5.2% (-8.2 to -2.2%)
18 Falls	18 Road injuries	-43% (-45.1 to -41.1%)	18 Other neoplasms	-1.1% (-5.4 to 2.5%)
19 Leukemia	19 Leukemia	-10.1% (-12.8 to -7.7%)	19 Leukemia	-6.3% (-9.4 to -3.2%)
20 Hypertensive heart disease	20 Other neoplasms	0.6% (-2.9 to 3.9%)	20 Liver cancer	-2.7% (-8.6 to 2.8%)
22 Other neoplasms	21 Liver cancer		21 Road injuries	
24 Liver cancer	24 Cardiomyopathy		23 Cardiomyopathy	

Legend:
Communicable, maternal, neonatal and nutritional
Non-communicable
Injuries

Appendix Figure 5b. Top 20 causes of death in Greece, Cyprus, and Western Europe, age groups 15-49 and 70+ with mean percent change

Top 20 causes of Deaths in Greece, Between 15 and 49 years, with mean perent change

Leading causes 2000	Leading causes 2010	Percent Change (counts) 2000 - 2010	Percent Change (rates) 2000 - 2010	Leading causes 2016	Percent Change (counts) 2010 - 2016	Percent Change (rates) 2010 - 2016
1 Road injuries	1 Ischemic heart disease	1.6% (-6.3 to 10.5%)	5.1% (-3.1 to 14.3%)	1 Ischemic heart disease	-12% (-22.4 to -1.2%)	-2.9% (-14.3 to 9%)
2 Ischemic heart disease	2 Road injuries	-30.9% (-35.5 to -25.7%)	-28.5% (-33.3 to -23.2%)	2 Road injuries	-22.8% (-31.4 to -13.6%)	-14.8% (-24.3 to -4.6%)
3 Lung cancer	3 Lung cancer	-17.3% (-27.4 to -5.9%)	-14.5% (-24.9 to -2.6%)	3 Lung cancer	-9.9% (-21.8 to 3.6%)	-0.5% (-13.7 to 14.4%)
4 Stroke	4 Stroke	-15.7% (-24.5 to -6.2%)	-12.9% (-22 to -3%)	4 Self-harm	-4% (-17.6 to 11.8%)	5.9% (-9 to 23.4%)
5 Drug use disorders	5 Drug use disorders	-6% (-20.7 to 11.7%)	-2.8% (-18 to 15.5%)	5 Stroke	-12.3% (-23 to -0.5%)	-3.2% (-15.1 to 9.8%)
6 Self-harm	6 Self-harm	5.5% (-9.3 to 21.5%)	9.1% (-6.2 to 25.7%)	6 Drug use disorders	-17.3% (-33.3 to 1.3%)	-8.7% (-26.4 to 11.8%)
7 Breast cancer	7 Breast cancer	-2.2% (-15.1 to 12.2%)	1.2% (-12.2 to 16%)	7 Breast cancer	-6.7% (-20.8 to 9.2%)	3% (-12.6 to 20.5%)
8 Brain cancer	8 Brain cancer	1.3% (-8.2 to 11%)	4.8% (-5 to 14.8%)	8 Brain cancer	-4.9% (-16.1 to 6.8%)	4.9% (-7.4 to 17.9%)
9 Falls	9 Drowning	-6.5% (-16.8 to 4.8%)	-3.3% (-14 to 8.4%)	9 Drowning	-17.3% (-27.8 to -5.8%)	-8.7% (-20.3 to 4%)
10 Drowning	10 Leukemia	-4.5% (-14.1 to 5.7%)	-1.2% (-11.1 to 9.3%)	10 Leukemia	-12.5% (-22.6 to -1.1%)	-3.4% (-14.6 to 9.2%)
11 Leukemia	11 Falls	-23% (-32.9 to -4.5%)	-20.4% (-30.6 to -1.2%)	11 Colorectal cancer	-4.1% (-16.6 to 10.5%)	5.9% (-8 to 22%)
12 Stomach cancer	12 Stomach cancer	-7.7% (-17.8 to 3.2%)	-4.5% (-15 to 6.7%)	12 Stomach cancer	-10% (-21 to 1.8%)	-0.6% (-12.8 to 12.3%)
13 Other neoplasms	13 Colorectal cancer	9.1% (-2.8 to 22.2%)	12.8% (0.6 to 26.4%)	13 Falls	-19.7% (-31 to -5.7%)	-11.4% (-23.8 to 4.1%)
14 Colorectal cancer	14 Interpersonal violence	5.6% (-14.4 to 28%)	9.3% (-11.5 to 32.4%)	14 Other neoplasms	-0.6% (-13.1 to 13.1%)	9.7% (-4.1 to 24.8%)
15 Lower respiratory infect	15 Lower respiratory infect	-9.2% (-21.8 to 5.1%)	-6.1% (-19.1 to 8.7%)	15 Interpersonal violence	-18.9% (-29.6 to -7.2%)	-10.5% (-22.3 to 2.5%)
16 Interpersonal violence	16 Other neoplasms	-14.1% (-22.9 to -3.9%)	-11.1% (-20.2 to -0.6%)	16 Lower respiratory infect	-9.5% (-22.3 to 5.9%)	-0.1% (-14.2 to 16.9%)
17 Chronic kidney disease	17 Pancreatic cancer	15.5% (2.1 to 30.4%)	19.4% (5.6 to 34.9%)	17 Pancreatic cancer	-5.5% (-17.3 to 7.4%)	4.3% (-8.8 to 18.6%)
18 Other cardiovascular	18 Cirrhosis alcohol	4.7% (-10.3 to 21.3%)	8.3% (-7.3 to 25.4%)	18 Cirrhosis alcohol	-7.5% (-23.1 to 11%)	2.1% (-15.1 to 22.4%)
19 Other transport inj	19 Chronic kidney disease	-28.3% (-36.1 to -19.4%)	-25.9% (-33.9 to -16.6%)	19 Chronic kidney disease	-3.8% (-16.1 to 10.4%)	6.2% (-7.4 to 21.9%)
20 Liver cancer	20 Ovarian cancer	2.2% (-15.1 to 22.5%)	5.7% (-12.1 to 26.7%)	20 Ovarian cancer	-8% (-24.2 to 10.7%)	1.5% (-16.4 to 22.1%)
21 Pancreatic cancer	21 Liver cancer			23 Liver cancer		
22 Cirrhosis alcohol	33 Other cardiovascular			28 Other cardiovascular		
25 Ovarian cancer	44 Other transport inj			43 Other transport inj		

Legend:
Communicable, maternal, neonatal and nutritional
Non-communicable
Injuries

Top 20 causes of Deaths in Greece, 70+ years, with mean perent change

Leading causes 2000	Leading causes 2010	Percent Change (counts) 2000 - 2010	Percent Change (rates) 2000 - 2010	Leading causes 2016	Percent Change (counts) 2010 - 2016	Percent Change (rates) 2010 - 2016
1 Ischemic heart disease	1 Ischemic heart disease	17.2% (11.4 to 23.5%)	-9.6% (-14.1 to -4.8%)	1 Ischemic heart disease	16.8% (9.3 to 24.5%)	10.3% (3.3 to 17.6%)
2 Stroke	2 Stroke	-13.7% (-19.7 to -7.3%)	-33.4% (-38 to -28.5%)	2 Stroke	16.8% (7.1 to 26.5%)	10.3% (1.2 to 19.5%)
3 Alzheimer disease	3 Alzheimer disease	34.6% (27.5 to 42.4%)	3.8% (-1.7 to 9.8%)	3 Alzheimer disease	31.9% (23.5 to 40.1%)	24.7% (16.7 to 32.3%)
4 Lung cancer	4 COPD	86.6% (73.7 to 100.7%)	43.9% (34 to 54.8%)	4 COPD	18.6% (10 to 29%)	12.1% (3.9 to 21.9%)
5 COPD	5 Lung cancer	13.1% (2.5 to 24.7%)	-12.8% (-20.9 to -3.8%)	5 Lung cancer	4% (-6 to 15%)	-1.8% (-11.1 to 8.6%)
6 Chronic kidney disease	6 Chronic kidney disease	0.8% (-6.8 to 9.7%)	-22.3% (-28.1 to -15.3%)	6 Chronic kidney disease	30.2% (18.9 to 42.4%)	23.1% (12.3 to 34.6%)
7 Lower respiratory infect	7 Colorectal cancer	23.9% (13.5 to 34.4%)	-4.4% (-12.5 to 3.7%)	7 Lower respiratory infect	29.4% (13.6 to 45.9%)	22.3% (7.3 to 37.9%)
8 Colorectal cancer	8 Lower respiratory infect	-1.6% (-12.7 to 11%)	-24.1% (-32.7 to -14.4%)	8 Colorectal cancer	13.4% (2.8 to 25.6%)	7.2% (-2.8 to 18.7%)
9 Other cardiovascular	9 Hypertensive heart disease	135.5% (17 to 210%)	81.7% (-9.7 to 139.1%)	9 Prostate cancer	20.5% (6.2 to 37.7%)	13.9% (0.3 to 30.1%)
10 Prostate cancer	10 Prostate cancer	18.6% (4.1 to 37.6%)	-8.5% (-19.7 to 6.1%)	10 Hypertensive heart disease	18.4% (6.5 to 32.1%)	11.9% (0.7 to 24.8%)
11 Liver cancer	11 Breast cancer	31.3% (17 to 48.6%)	1.3% (-9.8 to 14.6%)	11 Atrial fibrillation	39.3% (24.9 to 55.7%)	31.6% (18 to 47.1%)
12 Stomach cancer	12 Stomach cancer	6.8% (-2.5 to 15.6%)	-17.6% (-24.8 to -10.8%)	12 Breast cancer	11.1% (-2.3 to 26.6%)	5% (-7.7 to 19.6%)
13 Breast cancer	13 Liver cancer	-2.4% (-12.1 to 7.8%)	-24.7% (-32.2 to -16.9%)	13 Stomach cancer	6.4% (-3.2 to 16.5%)	0.5% (-8.5 to 10.1%)
14 Pancreatic cancer	14 Pancreatic cancer	34.5% (22.7 to 47.4%)	3.8% (-5.4 to 13.7%)	14 Pancreatic cancer	10.4% (-0.1 to 21.7%)	4.3% (-5.6 to 15%)
15 Leukemia	15 Atrial fibrillation	40.7% (24.7 to 57.1%)	8.5% (-3.8 to 21.1%)	15 Other cardiovascular	44.8% (30.5 to 61.6%)	36.8% (23.3 to 52.7%)
16 Bladder cancer	16 Leukemia	23.7% (12.8 to 35.9%)	-4.6% (-13 to 4.8%)	16 Liver cancer	-4.7% (-13.7 to 4.8%)	-10% (-18.5 to -0.9%)
17 Atrial fibrillation	17 Diabetes	28.5% (16.3 to 41.5%)	-0.9% (-10.3 to 9.1%)	17 Leukemia	9.2% (-1.2 to 20.7%)	3.1% (-6.6 to 14.1%)
18 Diabetes	18 Bladder cancer	13.3% (1.4 to 26.7%)	-12.6% (-21.8 to -2.3%)	18 Bladder cancer	15.1% (2.6 to 28.4%)	8.8% (-3.1 to 21.4%)
19 Cardiomyopathy	19 Other cardiovascular	-37.3% (-43.5 to -30.7%)	-51.6% (-56.4 to -46.6%)	19 Diabetes	11.4% (0.5 to 23.4%)	5.3% (-5.1 to 16.6%)
20 Hypertensive heart disease	20 Aortic aneurysm	29.2% (15.8 to 45.2%)	-0.3% (-10.6 to 12%)	20 Parkinson disease	23.8% (14.8 to 32.6%)	17% (8.5 to 25.3%)
21 Aortic aneurysm	21 Parkinson disease			21 Aortic aneurysm		
22 Parkinson disease	22 Cardiomyopathy			22 Cardiomyopathy		

Legend:
Communicable, maternal, neonatal and nutritional
Non-communicable
Injuries

Top 20 causes of Deaths in Cyprus, Between 15 and 49 years, with mean perent change

Leading causes 2000	Leading causes 2010	Percent Change (counts) 2000 - 2010	Percent Change (rates) 2000 - 2010	Leading causes 2016	Percent Change (counts) 2010 - 2016	Percent Change (rates) 2010 - 2016
1 Road injuries	1 Road injuries	-30.8% (-36.1 to -24.2%)	-44.7% (-49 to -39.4%)	1 Road injuries	-9.2% (-15.5 to -2.4%)	-16.3% (-22 to -10%)
2 Ischemic heart disease	2 Ischemic heart disease	-13.9% (-23.7 to -2.4%)	-31.2% (-39 to -22%)	2 Ischemic heart disease	-8.1% (-16.6 to 0.7%)	-15.3% (-23.1 to -7.1%)
3 Self-harm	3 Self-harm	1.5% (-11.7 to 19.6%)	-18.9% (-29.5 to -4.5%)	3 Self-harm	5.6% (-4.3 to 16.8%)	-2.6% (-11.7 to 7.7%)
4 Stroke	4 Breast cancer	9% (-10.9 to 30.7%)	-12.9% (-28.8 to 4.4%)	4 Breast cancer	1.7% (-11.8 to 16.1%)	-6.2% (-18.6 to 7.1%)
5 Breast cancer	5 Drug use disorders	21.3% (-7.4 to 53%)	-3.1% (-26 to 22.2%)	5 Drug use disorders	11.3% (-6.9 to 30.9%)	2.7% (-14.1 to 20.7%)
6 Other transport inj	6 Interpersonal violence	-7.5% (-21.7 to 10.9%)	-26.1% (-37.5 to -11.4%)	6 Interpersonal violence	1.5% (-9.1 to 13.9%)	-6.4% (-16.2 to 5.1%)
7 Interpersonal violence	7 Lung cancer	34.3% (12.8 to 60.7%)	7.3% (-9.9 to 28.3%)	7 Other transport inj	-2.1% (-14.7 to 12.5%)	-9.7% (-21.3 to 3.7%)
8 Drowning	8 Stroke	-25.7% (-35.9 to -14.2%)	-40.7% (-48.8 to -31.4%)	8 Lung cancer	-8.8% (-19.5 to 3.6%)	-15.9% (-25.8 to -4.5%)
9 Diabetes	9 Other transport inj	-18.6% (-32.9 to -2.4%)	-34.9% (-46.4 to -22.1%)	9 Stroke	-8.9% (-19.4 to 2.3%)	-16% (-25.6 to -5.6%)
10 Drug use disorders	10 Drowning	-15.3% (-25.8 to -2.6%)	-32.3% (-40.7 to -22.2%)	10 Drowning	-2.9% (-12.8 to 8.2%)	-10.5% (-19.6 to -0.3%)
11 Lung cancer	11 Diabetes	-12.3% (-24 to 1.2%)	-29.9% (-39.3 to -19.2%)	11 Diabetes	-5.1% (-14.9 to 4.9%)	-12.5% (-21.5 to -3.3%)
12 Leukemia	12 Brain cancer	14% (0.2 to 30.4%)	-9% (-19.9 to 4.1%)	12 Brain cancer	6.5% (-4.2 to 16.5%)	-1.8% (-11.6 to 7.4%)
13 Brain cancer	13 Leukemia	5.4% (-13.3 to 23.1%)	-15.8% (-30.7 to -1.6%)	13 Leukemia	1.8% (-6.5 to 12.1%)	-6.2% (-13.8 to 3.4%)
14 Mechanical forces	14 Other neoplasms	53.4% (15.4 to 82.1%)	22.6% (-7.8 to 45.5%)	14 Other neoplasms	8.2% (-2 to 20%)	-0.2% (-9.6 to 10.6%)
15 Cirrhosis alcohol	15 Falls	-3% (-15.5 to 12.1%)	-22.5% (-32.5 to -10.4%)	15 Endo/metab/blood/immune	3.6% (-7.1 to 15.1%)	-4.5% (-14.4 to 6.2%)
16 Endo/metab/blood/immune	16 Endo/metab/blood/immune	-4.9% (-18.6 to 15.7%)	-24.1% (-35 to -7.6%)	16 Falls	0.7% (-9.3 to 12.2%)	-7.1% (-16.3 to 3.5%)
17 Falls	17 Mechanical forces	-16.7% (-27.4 to -3.3%)	-33.5% (-42 to -22.7%)	17 Cirrhosis alcohol	-0.6% (-13.7 to 14.7%)	-8.4% (-20.4 to 5.8%)
18 Lymphoma	18 Cirrhosis alcohol	-12.6% (-26.3 to 4%)	-30.2% (-41.1 to -16.9%)	18 Mechanical forces	-5.8% (-15.4 to 4.1%)	-13.1% (-22 to -4%)
19 Other cardiovascular	19 Lymphoma	0.1% (-13.9 to 15.2%)	-20% (-31.2 to -8%)	19 Lymphoma	3.8% (-6.6 to 15.4%)	-4.3% (-13.9 to 6.4%)
20 Chronic kidney disease	20 Colorectal cancer	18.2% (1.1 to 38.5%)	-5.5% (-19.2 to 10.7%)	20 Colorectal cancer	-0.5% (-11.4 to 12.2%)	-8.2% (-18.3 to 3.5%)
21 Other neoplasms	21 Chronic kidney disease			21 Chronic kidney disease		
22 Colorectal cancer	22 Other cardiovascular			22 Other cardiovascular		

Legend:
Communicable, maternal, neonatal and nutritional
Non-communicable
Injuries

Top 20 causes of Deaths in Cyprus, 70+ years, with mean perent change

Leading causes 2000	Leading causes 2010	Percent Change (counts) 2000 - 2010	Percent Change (rates) 2000 - 2010	Leading causes 2016	Percent Change (counts) 2010 - 2016	Percent Change (rates) 2010 - 2016
1 Ischemic heart disease	1 Ischemic heart disease	-11.7% (-17.3 to -5%)	-32.6% (-36.8 to -27.4%)	1 Ischemic heart disease	11.6% (6.4 to 17.4%)	-2.6% (-7.1 to 2.4%)
2 Stroke	2 Alzheimer disease	27.3% (20.2 to 35.8%)	-2.8% (-8.2 to 3.7%)	2 Alzheimer disease	26.8% (22.1 to 31.6%)	10.7% (6.6 to 14.9%)
3 Alzheimer disease	3 Stroke	-14.5% (-23.6 to -4.1%)	-34.7% (-41.6 to -26.7%)	3 Stroke	10.9% (1.7 to 20.3%)	-3.2% (-11.2 to 5%)
4 Diabetes	4 Diabetes	-2.3% (-12.6 to 10.7%)	-25.4% (-33.3 to -15.5%)	4 Diabetes	10.4% (1.4 to 19.9%)	-3.7% (-11.5 to 4.6%)
5 COPD	5 COPD	2.8% (-6.8 to 13.7%)	-21.5% (-28.8 to -13.2%)	5 COPD	15.7% (7.4 to 24.9%)	1% (-6.2 to 9%)
6 Chronic kidney disease	6 Chronic kidney disease	11.7% (-0.4 to 24.5%)	-14.7% (-23.9 to -4.9%)	6 Chronic kidney disease	22.8% (13.2 to 33.2%)	7.2% (-1.1 to 16.3%)
7 Lower respiratory infect	7 Lung cancer	69.7% (45.6 to 96.5%)	29.5% (11.2 to 50.1%)	7 Lung cancer	7.5% (-3.2 to 18.2%)	-6.2% (-15.5 to 3.2%)
8 Other cardiovascular	8 Other cardiovascular	8.3% (-7.5 to 26.9%)	-17.3% (-29.4 to -3.1%)	8 Other cardiovascular	17.5% (4.5 to 30.2%)	2.6% (-8.8 to 13.6%)
9 Lung cancer	9 Lower respiratory infect	-10% (-23.8 to 3%)	-31.3% (-41.8 to -21.4%)	9 Lower respiratory infect	17.8% (3.7 to 30.9%)	2.9% (-9.5 to 14.3%)
10 Hypertensive heart disease	10 Prostate cancer	27.7% (0.5 to 54.1%)	-2.5% (-23.3 to 17.7%)	10 Prostate cancer	17% (4.1 to 30.1%)	2.2% (-9.1 to 13.6%)
11 Prostate cancer	11 Colorectal cancer	44.2% (27.4 to 63.3%)	10.1% (-2.7 to 24.7%)	11 Colorectal cancer	14.9% (5.7 to 25.6%)	0.3% (-7.8 to 9.6%)
12 Colorectal cancer	12 Hypertensive heart disease	3.5% (-13.5 to 27.1%)	-20.9% (-33.9 to -3%)	12 Hypertensive heart disease	19.2% (6.6 to 32.6%)	4% (-7 to 15.8%)
13 Atrial fibrillation	13 Breast cancer	41.5% (1.3 to 73.5%)	8.1% (-22.6 to 32.5%)	13 Urinary diseases	27.6% (16 to 41.5%)	11.4% (1.2 to 23.6%)
14 Falls	14 Urinary diseases	22% (6 to 40.7%)	-6.9% (-19 to 7.5%)	14 Breast cancer	15.4% (2.6 to 29.2%)	0.7% (-10.4 to 12.8%)
15 Urinary diseases	15 Atrial fibrillation	9.4% (-4.5 to 25.9%)	-16.5% (-27 to -3.9%)	15 Atrial fibrillation	25.6% (12.2 to 39.1%)	9.6% (-2.1 to 21.4%)
16 Aortic aneurysm	16 Falls	8.4% (-3.9 to 22.2%)	-17.2% (-26.6 to -6.7%)	16 Falls	22.8% (12.7 to 34.2%)	7.2% (-1.7 to 17.1%)
17 Breast cancer	17 Stomach cancer	35% (21.9 to 51.1%)	3.1% (-7 to 15.4%)	17 Stomach cancer	12% (2.6 to 21.1%)	-2.2% (-10.4 to 5.7%)
18 Stomach cancer	18 Pancreatic cancer	73.5% (54.2 to 96.3%)	32.5% (17.8 to 49.9%)	18 Pancreatic cancer	16.4% (5.6 to 28.2%)	1.6% (-7.8 to 11.9%)
19 Rheumatic heart disease	19 Parkinson disease	29% (18.5 to 39.8%)	-1.5% (-9.5 to 6.7%)	19 Parkinson disease	21.4% (16.8 to 26.2%)	6% (2 to 10.2%)
20 Road injuries	20 Leukemia	48.6% (0.8 to 82.9%)	13.5% (-23.1 to 39.7%)	20 Leukemia	17% (7.5 to 28.7%)	2.1% (-6.2 to 12.3%)
22 Parkinson disease	21 Aortic aneurysm			21 Aortic aneurysm		
24 Leukemia	26 Rheumatic heart disease			27 Rheumatic heart disease		
25 Pancreatic cancer	29 Road injuries			32 Road injuries		

Legend:
Communicable, maternal, neonatal and nutritional
Non-communicable
Injuries

Leading causes 2000	Leading causes 2010	Percent Change (counts) 2000 - 2010	Percent Change (rates) 2000 - 2010	Leading causes 2016	Percent Change (counts) 2010 - 2016	Percent Change (rates) 2010 - 2016
1 Self-harm	1 Self-harm	-16.2% (-23.7 to -9%)	-16.9% (-24.3 to -9.7%)	1 Self-harm	-8.9% (-14 to -3.7%)	-6.6% (-11.9 to -1.3%)
2 Road injuries	2 Road injuries	-45.7% (-48.6 to -43.4%)	-46.1% (-49 to -43.8%)	2 Road injuries	-19.3% (-22.5 to -15.8%)	-17.3% (-20.6 to -13.7%)
3 Ischemic heart disease	3 Ischemic heart disease	-27.2% (-30.5 to -23.9%)	-27.8% (-31 to -24.5%)	3 Ischemic heart disease	-16% (-20.1 to -11.9%)	-13.9% (-18.1 to -9.7%)
4 Lung cancer	4 Lung cancer	-16.4% (-20.3 to -12.4%)	-17% (-20.9 to -13.1%)	4 Lung cancer	-14.1% (-18.2 to -9.6%)	-11.9% (-16.1 to -7.3%)
5 Breast cancer	5 Breast cancer	-14.1% (-18.1 to -10.1%)	-14.8% (-18.8 to -10.8%)	5 Breast cancer	-9.5% (-14.2 to -4.1%)	-7.2% (-12.1 to -1.7%)
6 Stroke	6 Drug use disorders	-4% (-8.4 to 0.6%)	-4.8% (-9.1 to -0.2%)	6 Drug use disorders	0.2% (-8.2 to 5.6%)	2.7% (-5.9 to 8.2%)
7 Drug use disorders	7 Stroke	-30.9% (-33.9 to -27.8%)	-31.5% (-34.5 to -28.4%)	7 Stroke	-17.5% (-21 to -13.6%)	-15.4% (-19.1 to -11.5%)
8 Cirrhosis alcohol	8 Cirrhosis alcohol	-21.7% (-26.2 to -17.1%)	-22.3% (-26.8 to -17.8%)	8 Cirrhosis alcohol	-14.4% (-19.5 to -9.2%)	-12.3% (-17.6 to -7%)
9 HIV/AIDS	9 Colorectal cancer	-8.5% (-12.4 to -4.3%)	-9.2% (-13.1 to -5.1%)	9 Brain cancer	-4.6% (-11.7 to 0.4%)	-2.2% (-9.5 to 2.9%)
10 Alcohol use disorders	10 Brain cancer	-2% (-13.4 to 5%)	-2.8% (-14.1 to 4.1%)	10 Colorectal cancer	-8.1% (-12.4 to -2.8%)	-5.8% (-10.3 to -0.4%)
11 Colorectal cancer	11 Alcohol use disorders	-16.1% (-21.9 to -9.5%)	-16.8% (-22.5 to -10.2%)	11 Alcohol use disorders	-10.8% (-16.9 to -4.3%)	-8.6% (-14.9 to -1.9%)
12 Leukemia	12 Other neoplasms	-4.4% (-10.7 to 0.2%)	-5.1% (-11.4 to -0.6%)	12 Other neoplasms	-4.4% (-10.1 to 0.4%)	-2.1% (-7.9 to 2.9%)
13 Brain cancer	13 Cirrhosis hepatitis C	-18.2% (-23.1 to -12.8%)	-18.9% (-23.7 to -13.5%)	13 Leukemia	-11.6% (-16 to -7.7%)	-9.4% (-13.9 to -5.4%)
14 Cirrhosis hepatitis C	14 Leukemia	-20.8% (-25 to -17.3%)	-21.5% (-25.6 to -18%)	14 Cirrhosis hepatitis C	-13.1% (-18.7 to -7.5%)	-10.9% (-16.7 to -5.2%)
15 Other neoplasms	15 Falls	-21.6% (-26.9 to -10.8%)	-22.3% (-27.5 to -11.6%)	15 Falls	-13.4% (-17.7 to -8.5%)	-11.3% (-15.6 to -6.2%)
16 Falls	16 HIV/AIDS	-46.2% (-47.6 to -44.9%)	-46.6% (-48 to -45.3%)	16 Stomach cancer	-14.1% (-18 to -10%)	-12% (-16 to -7.7%)
17 Stomach cancer	17 Stomach cancer	-20.5% (-24 to -16.8%)	-21.1% (-24.6 to -17.5%)	17 Other cardiovascular	-10.7% (-15.4 to -5.6%)	-8.5% (-13.3 to -3.2%)
18 Lower respiratory infect	18 Other cardiovascular	-14.7% (-20.1 to -9.7%)	-15.4% (-20.7 to -10.4%)	18 Pancreatic cancer	-5.9% (-10.9 to -1%)	-3.6% (-8.7 to 1.4%)
19 Interpersonal violence	19 Lower respiratory infect	-22.5% (-26.7 to -18%)	-23.2% (-27.2 to -18.6%)	19 Lower respiratory infect	-12.5% (-16.9 to -7.1%)	-10.3% (-14.9 to -4.8%)
20 Other cardiovascular	20 Pancreatic cancer	6.8% (1.4 to 12.2%)	6% (0.6 to 11.3%)	20 Interpersonal violence	-13.7% (-17.7 to -8.8%)	-11.5% (-15.6 to -6.5%)
23 Pancreatic cancer	21 Interpersonal violence			23 HIV/AIDS		

Legend:
Communicable, maternal, neonatal and nutritional
Non-communicable
Injuries

Top 20 causes of Deaths in Western Europe, 70+ years, with mean perent change

Leading causes 2000	Leading causes 2010	Percent Change (counts) 2000 - 2010	Percent Change (rates) 2000 - 2010	Leading causes 2016	Percent Change (counts) 2010 - 2016	Percent Change (rates) 2010 - 2016
1 Ischemic heart disease	1 Ischemic heart disease	-14.4% (-16.5 to -11.9%)	-28.9% (-30.6 to -26.9%)	1 Ischemic heart disease	7.2% (4.4 to 10%)	-2.8% (-5.4 to -0.3%)
2 Alzheimer disease	2 Alzheimer disease	23.7% (20.2 to 27.1%)	2.8% (-0.2 to 5.5%)	2 Alzheimer disease	20.8% (17.9 to 23.8%)	9.6% (6.8 to 12.3%)
3 Stroke	3 Stroke	-11.2% (-14.2 to -8.1%)	-26.2% (-28.7 to -23.7%)	3 Stroke	6% (2.8 to 9.3%)	-3.9% (-6.8 to -1%)
4 COPD	4 COPD	9.2% (6.3 to 13.2%)	-9.3% (-11.7 to -6%)	4 COPD	10.1% (7.1 to 12.8%)	-0.2% (-2.9 to 2.3%)
5 Lung cancer	5 Lung cancer	16.6% (13.1 to 20.2%)	-3.2% (-6.1 to -0.2%)	5 Lung cancer	4.9% (1.5 to 8.6%)	-4.9% (-8 to -1.6%)
6 Lower respiratory infect	6 Lower respiratory infect	0.9% (-3.1 to 4.9%)	-16.2% (-19.5 to -12.9%)	6 Lower respiratory infect	14.3% (9.7 to 18.9%)	3.6% (-0.6 to 7.8%)
7 Colorectal cancer	7 Colorectal cancer	11% (7.5 to 14.9%)	-7.8% (-10.7 to -4.6%)	7 Colorectal cancer	8.1% (3.8 to 12.9%)	-2% (-5.9 to 2.4%)
8 Other cardiovascular	8 Other cardiovascular	9.9% (6 to 14.2%)	-8.7% (-12 to -5.2%)	8 Other cardiovascular	12.4% (7.9 to 17.5%)	1.9% (-2.2 to 6.5%)
9 Diabetes	9 Diabetes	5% (0.9 to 9.1%)	-12.8% (-16.2 to -9.4%)	9 Chronic kidney disease	16.6% (12.7 to 20.6%)	5.7% (2.2 to 9.3%)
10 Prostate cancer	10 Chronic kidney disease	51.2% (46.3 to 56.3%)	25.5% (21.5 to 29.8%)	10 Diabetes	6.9% (2.8 to 11.1%)	-3.1% (-6.8 to 0.7%)
11 Stomach cancer	11 Prostate cancer	12.6% (7 to 32.7%)	-6.5% (-11.1 to 10.2%)	11 Prostate cancer	17.7% (11.9 to 23.2%)	6.7% (1.5 to 11.7%)
12 Chronic kidney disease	12 Hypertensive heart disease	34.8% (2.4 to 50.5%)	12% (-15 to 25%)	12 Hypertensive heart disease	13.8% (6.8 to 21.3%)	3.2% (-3.2 to 10%)
13 Breast cancer	13 Atrial fibrillation	17.9% (13.3 to 22.7%)	-2.1% (-5.9 to 1.9%)	13 Atrial fibrillation	18.5% (13.6 to 23.1%)	7.5% (3 to 11.6%)
14 Hypertensive heart disease	14 Breast cancer	9.4% (4.9 to 13.7%)	-9.2% (-12.9 to -5.5%)	14 Falls	15.5% (11.1 to 19.6%)	4.7% (0.7 to 8.4%)
15 Atrial fibrillation	15 Pancreatic cancer	32.9% (28.2 to 37.9%)	10.4% (6.5 to 14.5%)	15 Pancreatic cancer	10.9% (6.1 to 15.2%)	0.5% (-3.8 to 4.4%)
16 Cardiomyopathy	16 Falls	21.5% (8.3 to 28.1%)	0.9% (-10.1 to 6.4%)	16 Breast cancer	4.6% (-0.4 to 9.3%)	-5.2% (-9.7 to -0.9%)
17 Falls	17 Stomach cancer	-6.1% (-9.1 to -3.3%)	-22% (-24.5 to -19.7%)	17 Stomach cancer	5% (1.4 to 8.5%)	-4.8% (-8 to -1.6%)
18 Pancreatic cancer	18 Cardiomyopathy	-11% (-19.4 to 26%)	-26.1% (-33.1 to 4.6%)	18 Cardiomyopathy	16.9% (11.9 to 21.9%)	6% (1.5 to 10.6%)
19 Aortic aneurysm	19 Parkinson disease	38% (29.9 to 45.6%)	14.6% (7.9 to 20.9%)	19 Parkinson disease	12.4% (9.2 to 15.4%)	1.9% (-1 to 4.6%)
20 Bladder cancer	20 Bladder cancer	17.9% (13.8 to 22.2%)	-2.1% (-5.5 to 1.5%)	20 Bladder cancer	13.3% (9 to 18.1%)	2.7% (-1.2 to 7%)
22 Parkinson disease	24 Aortic aneurysm			24 Aortic aneurysm		

Legend:
Communicable, maternal, neonatal and nutritional
Non-communicable
Injuries

Appendix Figure 6. Health expenditure compared to mortality in Greece and Cyprus

Cyprus Government health expenditure (GHE), Out of pocket health expenditure (OOP), Prepaid public health expenditure (PPP), and total health expenditure (THE) from 2000 to 2014 compared to mortality rate (all ages) and age-standardised mortality rate

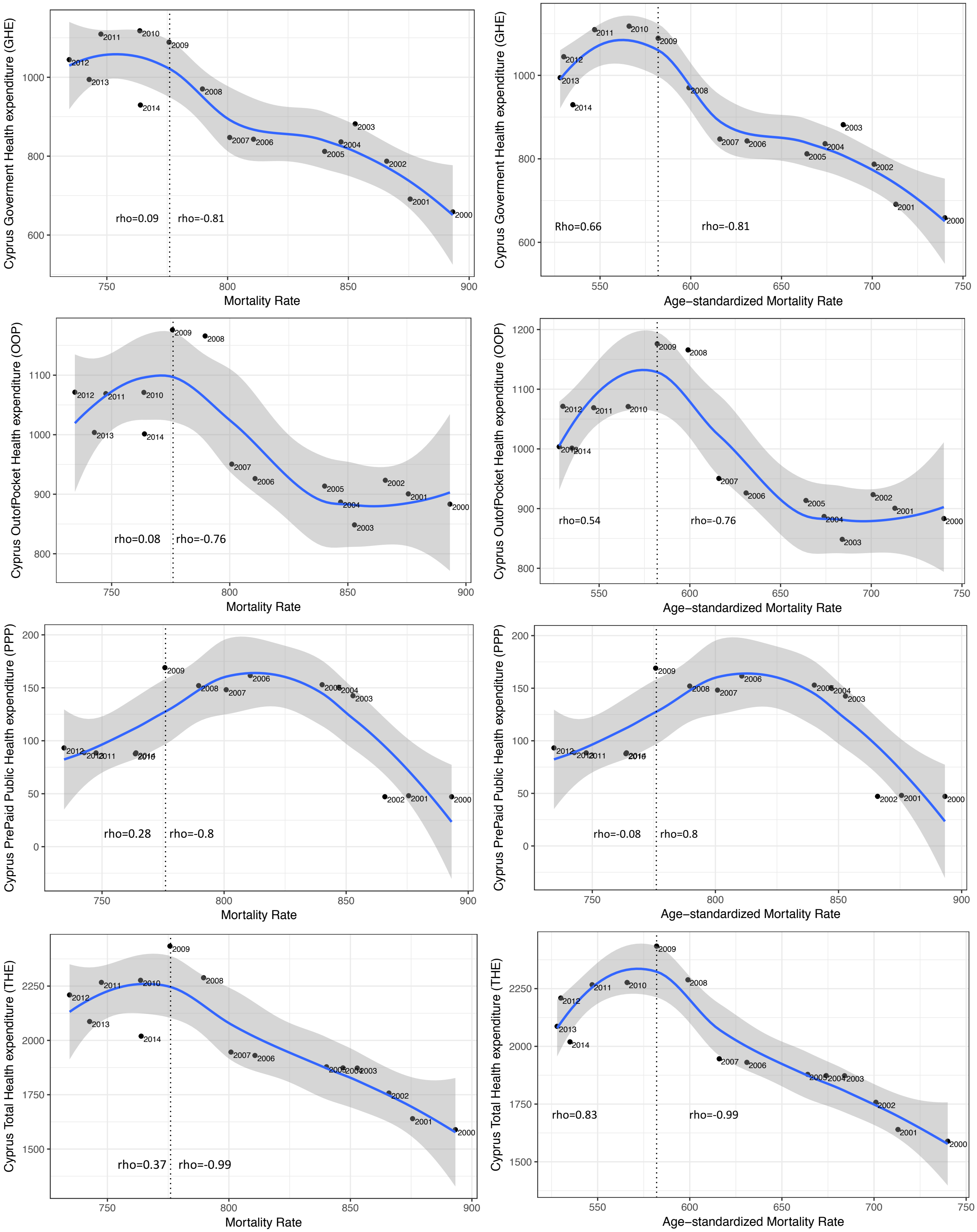

Greece Government health expenditure (GHE), Out of pocket health expenditure (OOP), Prepaid public health expenditure (PPP), and total health expenditure (THE) from 2000 to 2014 compared to mortality rate (all ages) and age-standardised mortality rate

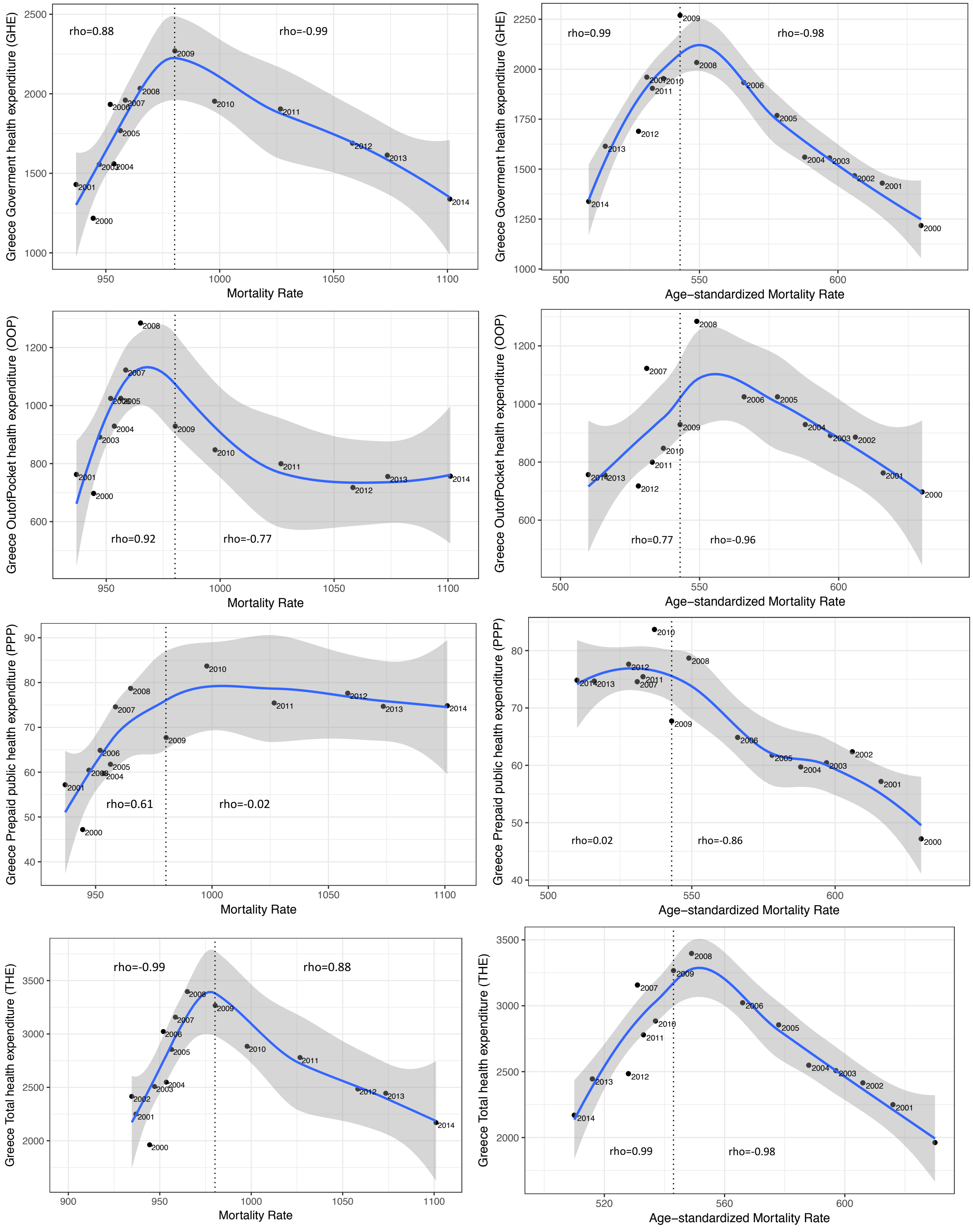

Appendix Figure 7. Top-ranked causes of years lived with disability (YLDs) at all ages and both sexes in Greece, Cyprus, and Western Europe for 2000 and 2016

Both sexes, All ages, 2000, YLDs per 100,000

	Greece	Cyprus	W Europe
Low back & neck pain	1	1	1
Sense organ diseases	2	4	2
Migraine	3	3	4
Depressive disorders	4	5	5
Skin diseases	5	2	3
Anxiety disorders	6	6	6
Oral disorders	7	9	8
Falls	8	8	7
COPD	9	28	22
Road injuries	10	12	14
Other musculoskeletal	11	10	10
Diabetes	12	7	9
Cerebrovascular disease	13	16	13
Bipolar disorder	14	13	17
Osteoarthritis	15	15	12
Asthma	16	11	11
Schizophrenia	17	17	19
Ischemic heart disease	18	19	20
Other cardiovascular	19	14	15
Other mental & substance	20	18	21
Alzheimer disease	21	21	18
Drug use disorders	22	22	16
Autistic spectrum	23	23	24
Tension headache	24	25	28
Congenital defects	25	20	26

Both sexes, All ages, 2016, YLDs per 100,000

	Greece	Cyprus	W Europe
Low back & neck pain	1	1	1
Sense organ diseases	2	4	2
Migraine	3	2	4
Depressive disorders	4	5	5
Skin diseases	5	3	3
Anxiety disorders	6	6	6
Oral disorders	7	9	8
Falls	8	8	7
COPD	9	29	22
Cerebrovascular disease	10	15	13
Other musculoskeletal	11	10	10
Diabetes	12	7	9
Osteoarthritis	13	13	11
Alzheimer disease	14	19	14
Road injuries	15	14	17
Bipolar disorder	16	12	16
Ischemic heart disease	17	20	21
Asthma	18	11	12
Schizophrenia	19	17	19
Other mental & substance	20	18	20
Other cardiovascular	21	16	15
Drug use disorders	22	21	18
Chronic kidney disease	23	32	28
Atrial fibrillation	24	34	23
Tension headache	25	23	29

IHME

Appendix Figure 8. YLDs per 100,000 attributable to metabolic and behavioural risk factors in Greece, Cyprus, Western Europe, and globally, 2000–2016

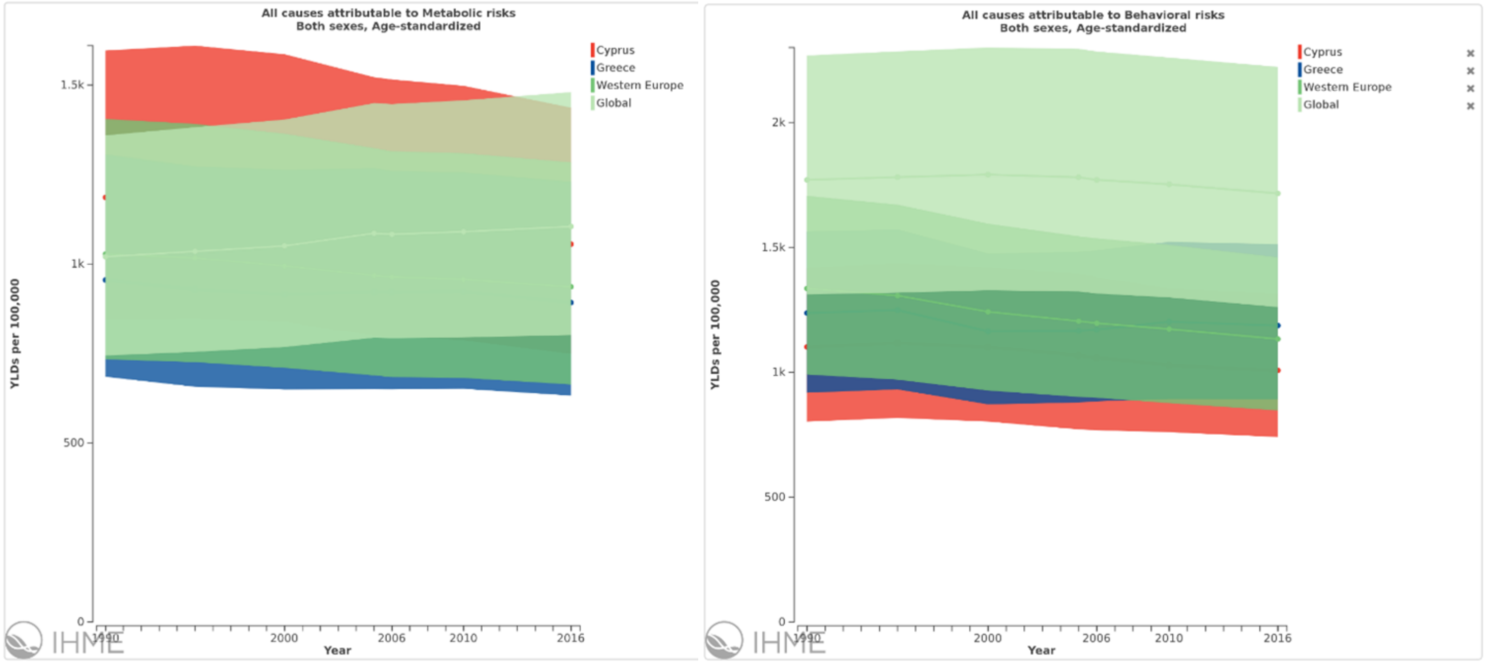

Supplement: Supplementary appendix [file mmc1.pdf]
